# Diindolocarbazole as a Core Structure for Narrow‐Emitting and Highly Efficient Blue Organic Light‐Emitting Diodes

**DOI:** 10.1002/advs.202504625

**Published:** 2025-06-10

**Authors:** Jihoon Kang, Junyoung Moon, Soon Ok Jeon, Unhyeok Jo, Seungwon Han, Sangmo Kim, Jun Yeob Lee

**Affiliations:** ^1^ School of Chemical Engineering Sungkyunkwan University 2066, Seobu‐ro, Jangan‐gu Suwon Gyeonggi 16419 Republic of Korea; ^2^ Samsung Advanced Institute of Technology Samsung Electronics Co., Ltd. 130 Samsung‐ro, Yeongtong‐gu Suwon Gyeonggi 16678 Republic of Korea; ^3^ Department of Display Convergence Engineering Sungkyunkwan University 2066, Seobu‐ro, Jangan‐gu Suwon Gyeonggi 16419 Republic of Korea; ^4^ SKKU Institute of Energy Science and Technology Sungkyunkwan University 2066, Seobu‐ro, Jangan‐gu Suwon Gyeonggi 16419 Republic of Korea

**Keywords:** blue emitter, fluorescence, multiple resonance, narrow emission

## Abstract

In this study, a molecular design approach inducing high efficiency and narrow emission in blue organic light‐emitting diodes (OLEDs) using indolocarbazole and diindolocarbazole backbone structures are proposed. The indolocarbazole and diindolocarbazole backbone structures are used as a core structure and the indole is protected with spiro configured fluorene for rigidity and suppressed intermolecular interaction. The indolocarbazole and diindolocarbazole derived compounds show multiple resonance–thermally activated delayed fluorescence emission even without any electron deficient unit within the molecular structure and narrow emission spectrum with a full width at half maximum (FWHM) of less than 20 nm. As a result, the blue OLEDs fabricated using the unique diindolocarbazole emitter delivered high external quantum efficiency of 28.2% and a sharp emission spectrum with a FWHM of 20 nm.

## Graphical abstract

A molecular design approach inducing high efficiency and narrow emission in blue organic light‐emitting diodes using indolocarbazole and diindolocarbazole backbone structures realized high external quantum efficiency of 28.2% and sharp emission spectrum with a full width at half maximum of 15 nm.

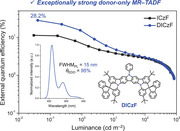

## 1 Introduction

High external quantum efficiency (EQE) and high color purity have been key performances of organic light‐emitting diodes (OLEDs) to reduce power consumption and increase the color gamut of display panels.^[^ ^1^, ^2^, ^3^, ^4^, ^5^ ^]^ Therefore, a lot of researches about light‐emitting materials have been directed to develop highly efficient and narrow‐emitting compounds to realize the desired device performances.^[^ ^6^, ^7^, ^8^, ^9^, ^10^, ^11^, ^12^, ^13^, ^14^, ^15^ ^]^ In particular, the development of narrow‐emitting blue compounds has been a main interest in the OLED community.

One of the most effective approaches for narrow emission is to utilize multiple resonance (MR)–thermally activated delayed fluorescence (TADF) emitters which emit by short range charge transfer (SRCT) mechanism through atomically separated the highest occupied molecular orbital (HOMO) and the lowest unoccupied molecular orbital (LUMO).^[^ ^16^, ^17^, ^18^, ^19^ ^]^ Representative MR–TADF emitters include boron‐nitrogen, boron‐oxygen, and nitrogen‐carbonyl compounds with strong SRCT characters which rigidify the molecular structure in excited state and increase oscillator strength for extensive orbital overlap.^[^ ^20^, ^21^, ^22^, ^23^, ^24^, ^25^, ^26^, ^27^ ^]^ As a result, most MR–TADF emitters allowed a small full width at half maximum (FWHM) less than 30 nm and photoluminescence quantum yield (PLQY) higher than 90%.^[^ ^28^, ^29^, ^30^, ^31^, ^32^, ^33^, ^34^, ^35^ ^]^ The small FWHM and high PLQY also enabled high EQE and narrow electroluminescence (EL) spectrum in the OLEDs.

Other than the strong SRCT based compounds, fused indolocarbazole‐based emitters have also realized high efficiency and high color purity as donor based TADF compounds through a nitrogen.^[^ ^8^, ^10^, ^12^, ^36^, ^37^, ^38^, ^39^ ^]^ Although the SRCT of them was not as strong as that of boron derivatives, the donor based fused indolocarbazole compounds delivered narrow emission through MR structure and TADF behavior via spin‐vibronic coupling assisted up‐conversion process. The fused indolocarbazole was also incorporated as a weak donor playing a role of acceptor in the donor–acceptor type TADF compounds although MR characters were lost.^[^ ^40^ ^]^ Similarly, donor based hexacarbazolylbenzene employing a 3,6‐dimethoxy‐9*H*‐carbazole moiety performed as TADF emitters.^[^ ^41^ ^]^ However, the emission bandwidth of hexacarbazole and indolocarbazole based donor–acceptor compounds was large and PLQY was quite low in spite of TADF property because of flexible molecular structure. Recent work reported rigid spiro‐indolo[3,2,1‐*de*]acridine moiety based fluorescent emitters with spirofluorene configured backbone structure for improved PLQY and narrow emission.^[^ ^42^ ^]^ However, TADF mechanism was not activated for triplet exciton utilization. Therefore, further development of new donor based core structures for efficient and sharp emission in the blue OLEDs while keeping the MR structure is necessary to achieve both high efficiency and narrow emission using donor only main skeleton.

In this study, a novel molecular design concept realizing both high EQE and small FWHM in the blue OLEDs was developed using 5,11‐diphenyl‐5,11‐dihydroindolo[3,2‐*b*]carbazole (ICz) and 5,8,14‐triphenyl‐8,14‐dihydro‐5*H*‐pyrrolo[3,2‐*b*:4,5‐*b*']dicarbazole (DICz) backbone structures fused with a spirofluorene unit through a six‐membered ring structure. Combination of aforementioned chemical moieties provided the strong donor based blue MR–TADF emitters, ICzF and DICzF. The ICzF and DICzF compounds have a backbone structure with a central carbazole fused with one or two indole units and protected with spiro configured fluorene unit for rigidity and suppressed intermolecular interactions. The DICz derived DICzF showed better performances than ICz derived ICzF, showing high PLQY of 86% and small FWHM of 15 nm in solution as a blue TADF emitter. In the device application, a high EQE of 28.2% and small FWHM of 20 nm were achieved using the DICzF emitter. This is the first demonstration of MR–TADF emission using strongly electron rich diindolocarbazole unit even without any electron deficient units in the molecular structure. This work opens a new way of enabling TADF emission in organic emitters using only electron rich units in the molecular structure.

## 2 Results and Discussion

### 2.1 Molecular Design and Simulation

Main backbone structures of the emitter employed in this work are ICz and DICz which have a carbazole fused with one or two indole units, respectively. The design of the emitter was inspired by the MR character of the fused indolocarbazole‐based compounds enabling narrow emission. As exemplified in 2,5,11,14‐tetra‐*tert*‐butyl‐indolo[3,2,1‐*jk*]‐indolo[1′,2′,3′:1,7]indolo[3,2‐*b*]carbazole (tBisICz), many fused indolocarbazole‐based derivatives demonstrated narrow emission through the MR nature because of nitrogen managing the orbital distribution.^[^ ^12^, ^37^, ^38^, ^39^ ^]^ The nitrogen atoms embedded in those materials induced alternating HOMO and LUMO distribution for SRCT in the MR–TADF emitters. In the case of ICz backbone, the nitrogen atoms may behave as an electron rich unit and can manage orbital distribution like boron and nitrogen in the conventional boron‐nitrogen based MR‐type materials. As the key parameter for the MR characteristics is SRCT through alternating HOMO and LUMO distribution, a molecular design employing an electron rich unit can also be used to develop MR–TADF emitters.

**Figure 1:**
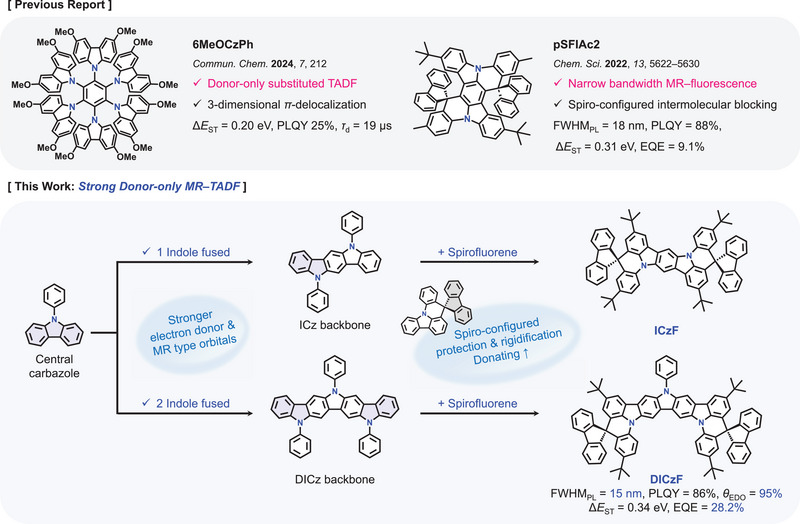
The molecular design concept of strong donor‐only MR–TADF emitters containing spirofluorene‐configured indolo[3,2,1‐*de*]acridine moiety.

Several indolocarbazole backbone structures are available, but only ICz enabling alternating HOMO and LUMO distribution can be used as the backbone structure of the MR–TADF emitters. Figure S13a–e (Supporting Information) compares the HOMO and LUMO distribution of the indolocarbazole backbone structures with different ring condensation arrangements. Among them, only indolo[3,2‐*b*]carbazole delivers alternating HOMO and LUMO distributions suitable for MR–TADF emission. Therefore, it can be a candidate as a core structure of the MR–TADF emitters. Moreover, DICz with an additional fused indole unit in the ICz core also exhibits alternating HOMO and LUMO and performs as a core structure of the MR–TADF emitter (Figure S13f, Supporting Information). The ICz and DICz were hired as the core structure and they were protected with spiro configured fluorene unit for rigidity and suppressed intermolecular interactions, providing ICzF and DICzF as target compounds. The spirofluorene‐configured indoloacridine structure in the target compounds not only enhances donor strength by restricting local conjugation but also introduces a large dihedral angle that provides intermolecular blocking function (Figure S14, Supporting Information). The design motivation of the emitters is illustrated in **Figure** **1**.

**Figure 2:**
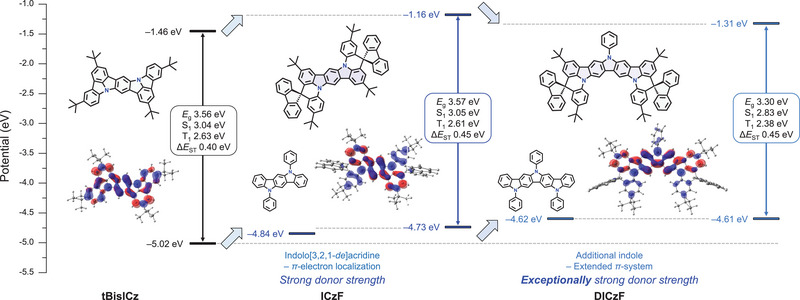
DFT calculation data of ICzF and DICzF compared to tBisICz. The energy level of HOMO and LUMO, energy gap, S_1_ and T_1_ excitation energies, and Δ*E*_ST_ are provided. Blue filled region represents HOMO and red filled region represents LUMO distribution, respectively.

The quantum chemical properties of ICzF and DICzF were predicted using density functional theory (DFT) simulations at the B3LYP/6‐31G(*d*) level with the Gaussian 16 software.^[^ ^43^, ^44^, ^45^, ^46^, ^47^ ^]^ The ground state, singlet, and triplet excited state properties were simulated via single‐point calculations after optimizing the S_0_, S_1_, and T_1_ geometries using the DFT and time dependent‐DFT methods, respectively. To compare the calculated properties of ICzF and DICzF, tBisICz was selected as an indolo[3,2,1‐*jk*]carbazole‐based MR–TADF reference material, and the corresponding calculation results are presented in **Figure** **2**. The predicted material properties are listed in Table S1 (Supporting Information).

Compared to the fully conjugated tBisICz structure, the introduction of an indolo[3,2,1‐*de*]acridine moiety in ICzF significantly increased its electron donating properties due to the *π*‐electron localization effect. This change is evident in the shallow HOMO energy level (*E*_HOMO_) of ICzF (tBisICz: −5.02 eV; ICzF: −4.73 eV). Since the *π*‐electron localization did not affect the bandgap energy (*E*_g_) of the molecules, the LUMO energy level (*E*_LUMO_) of ICzF was destabilized along with its *E*_HOMO_. Additionally, both molecules were predicted to have nearly identical S_1_ and T_1_ excitation energies, suggesting blue TADF emissions. DICzF, which has an extended molecular structure due to the additional condensation of an indole group compared to ICzF, led to conjugation extension and enhanced electron donating properties, resulting in a shallow *E*_HOMO_ of −4.61 eV. The extended conjugation length of DICzF stabilized the *E*_g_ as well as the S_1_ and T_1_ excitation energies.

Comparing the HOMO–LUMO distributions of tBisICz, ICzF, and DICzF, it is evident that ICzF and DICzF maintain nonbonding orbital characteristics (i.e., MR type orbital separation). In particular, the HOMO distributions remain nearly identical, while the LUMO distributions exhibit localized patterns on the ICz and DICz backbones, except for the side phenyl groups, due to *π*‐electron localization. A similar MR type orbital pattern is also observed in the natural transition orbital (NTO) distributions in the radiative S_1_ excited state (Figures S15 and S16, Supporting Information). The reorganization energy (*λ*_Reorg_) between the S_0_ and S_1_ states, obtained from vertical absorption and vertical emission calculations, was determined to be 0.2248 , 0.1970 , and 0.1476 eV for tBisICz, ICzF, and DICzF, respectively. A smaller *λ*_Reorg_ indicates a more rigidified molecular geometry, which restricts geometrical relaxation during the S_1_–S_0_ state transition and is advantageous for achieving a narrow‐bandwidth emission spectrum.^[^ ^48^ ^]^ This improvement in *λ*_Reorg_ is attributed to the inherent structural advantage of the indolo[3,2,1‐*de*]acridine moiety, suggesting that ICzF and DICzF are capable of realizing narrow‐bandwidth emission. As a result, ICzF and DICzF were predicted to possess MR emitter type narrow‐bandwidth blue emission.

To leverage the *sp* ^3^ C‐centered blocking position in the indolo[3,2,1‐*de*]acridine moiety, two spirofluorene groups are introduced into ICzF and DICzF. This modification aimed to increase the molecular volume, control the intermolecular distance, and prevent solid state *π*–*π* stacking.^[^ ^49^ ^]^ Generally, the blocking function is most effective when the bulky substituent is introduced along the z‐axis (i.e., perpendicular to the main backbone structure), ensuring a sufficient dihedral angle.^[^ ^37^, ^50^, ^51^, ^52^, ^53^ ^]^ The dihedral angle between the spirofluorene group and the (D)ICz backbone in the optimized S_0_ geometry of both ICzF and DICzF was calculated to be 93.4°, indicating a nearly perpendicular arrangement (Figure S17, Supporting Information). Additionally, the molecular cubic volumes (*V*_cubic_) of ICzF and DICzF, calculated using Multiwfn software, were 2.50 nm^3^ and 3.31 nm^3^, respectively, representing a significant increase compared to that of tBisICz (1.12 nm^3^) (Figure S18, Supporting Information).^[^ ^54^ ^]^ Consequently, ICzF and DICzF are theoretically predicted to exhibit strong electron‐donating characteristics, maintain MR‐type narrow‐bandwidth blue emission, and effectively control intermolecular distances.

### 2.2 Material Characterization

**Scheme 1:**
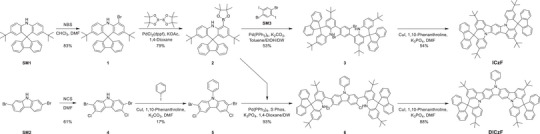
Synthesis scheme of ICzF and DICzF.

The synthesis scheme for the ICzF and DICzF MR‐TADF emitters is described in **Scheme** **1**. Detailed information regarding the synthetic methods and analytical data is provided in the Synthesis section of the Supporting Information (Figures S1–S12, Supporting Information). The starting material of the common intermediate 2, 2,7‐di‐*tert*‐butyl‐10*H*‐spiro[acridine‐9,9′‐fluorene] (SM1), was synthesized via a facile direct cyclization reaction from bis(4‐(*tert*‐butyl)phenyl)amine.^[^ ^55^ ^]^ Bromination was conducted to functionalize the *ortho*‐position of the nitrogen in SM1, followed by the introduction of a pinacol ester group under Miyaura borylation conditions to obtain intermediate 2. The intermediate 4, 2,7‐dibromo‐3,6‐dichloro‐9*H*‐carbazole, was synthesized via chlorination of SM2. Subsequently, the ICz‐ and DICz‐derived target materials, ICzF and DICzF, were obtained via a stepwise approach involving Suzuki–Miyaura cross‐coupling and C–N cyclization under Ullmann conditions. The target materials were first purified through column chromatography and recrystallization, followed by train sublimation to ensure high purity before material and device characterization.

**Figure 3:**
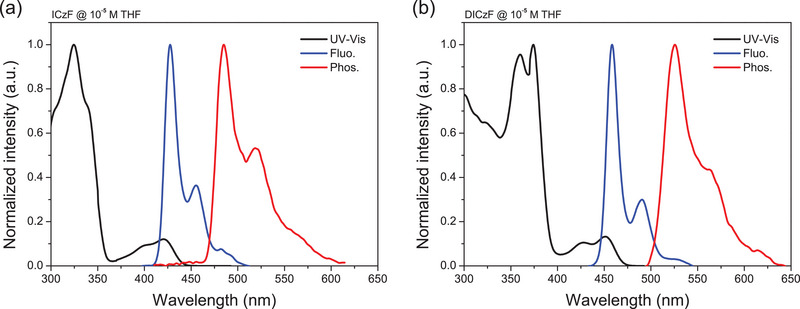
Solution PL analysis of (a) ICzF and (b) DICzF measured in 10^−5^ m tetrahydrofuran (THF) solution. UV–vis absorption spectra (black) was measured at room temperature, and fluorescence (Fluo.; blue) and phosphorescence (Phos.; red) spectra were measured at 77K.

Absorption and emission characteristics of the ICzF and DICzF compounds were collected at room temperature using diluted tetrahydrofuran and toluene solutions, respectively. The ultraviolet–visible (UV–vis) absorption spectra and steady‐state photoluminescence (PL) spectra are illustrated in **Figure** **3**, and material characterization data are summarized in **Table** **1**. The UV–vis spectra featured strong *π*–*π* ^*^ transition of the backbone structure at below 360 nm in the ICzF and at below 400 nm in the DICzF. The overall absorption band of DICzF was observed to be red‐shifted by ≈30 nm compared to that of ICzF, due to the extended conjugated structure through the additional indole unit. Relatively weak SRCT absorption was recorded at ≈420 and 451 nm in ICzF and DICzF by MR character of the core structures, respectively. The MR character was reflected on the sharp PL emission profile in diluted tetrahydrofuran solution with a peak wavelength of PL emission (*λ*_PL_)/FWHM_PL_ of 428 nm/16 nm (ICzF) and 459 nm/15 nm (DICzF) at 77K, respectively. In the toluene solution, *λ*_PL_/FWHM_PL_ was measured to be 436 nm/22 nm (ICzF) and 465 nm/19 nm (DICzF) at room temperature, respectively (Figure S19, Supporting Information). The *λ*_PL_ was red‐shifted but the FWHM was reduced in the DICzF emitter relative to the ICzF emitter. The PL spectrum shift is caused by the extended conjugation structure, while the small FWHM is due to the rigidified molecular structure through the fused indole unit. This trend agrees well with the computed *λ*_Reorg_ values. The *λ*_PL_ of the emitters was shifted to short wavelength and the FWHM was decreased by suppressed vibrational motion at 77K. The Stokes shift was only 10 nm in the ICzF and 8 nm in the DICzF TADF emitters. Singlet‐triplet energy gaps (Δ*E*_ST_) were 0.33 eV for ICzF and 0.34 eV for DICzF from the singlet energy (*E*_S_) in the fluorescence spectrum and triplet energy (*E*_T_) in the phosphorescence spectrum at 77K.

**Table 1:** Summarized material characterization data of ICzF and DICzF.

	*E*_HOMO_ ^a)^	*E*_LUMO_ ^b)^	*E*_opt_ ^c)^	λ_abs_ ^d)^	λ_PL_ ^e)^	FWHM^e)^	*E*_s_ ^e)^	*E*_T_ ^e)^	Δ*E*_ST_ ^h)^	Stokes shift^i)^
	[eV]	[eV]	[eV]	[nm]	[nm]	[nm]	[eV]	[eV]	[eV]	[nm]
ICzF	−5.50	−2.68	2.82	420	428^f)^;436^g)^	16^f)^;22^g)^	2.90^f)^	2.55^f)^	0.33	10
DICzF	−5.41	−2.77	2.64	451	459^f)^;465^g)^	15^f)^;19^g)^	2.70^f)^	2.36^f)^	0.34	8

^a)^ Measured value from onset voltage of oxidation scan;

^b)^ Estimated by *E*_opt_ and *E*_HOMO_ (*E*_LUMO_ = *E*_HOMO_ + *E*_opt_);

^c)^ Calculated from the edge position of absorption spectrum;

^d)^ The largest peak wavelength of absorption bands;

^e)^ Fluorescence and phosphorescence emission were measured without and with 1 ms delay;

^f)^ Measured in diluted tetrahydrofuran solution at 77K;

^g)^ Measured in diluted toluene solution at room temperature;

^h)^ Δ*E*_ST_ = *E*_S_ – *E*_T_;

^i)^ Gap between the largest wavelength absorption peak and peak singlet emission wavelength.

HOMO and LUMO levels were recorded using an electrochemically determined HOMO and optical bandgap (*E*_opt_) from the edge position of UV–vis absorption band. The *E*_HOMO_ was −5.50 eV for ICzF and −5.41 eV for DICzF from the cyclic voltammetry analysis (Figure S20, Supporting Information). The *E*_LUMO_ were estimated as −2.68 eV for ICzF and −2.77 eV for DICzF. Indole‐extended conjugation structure of DICzF provided shallow *E*_HOMO_ and deep *E*_LUMO_ compared to ICzF.

To determine the thermal decomposition temperature (*T*_d_) of ICzF and DICzF, thermogravimetric analysis (TGA) was performed under N_2_ conditions (Figure S21, Supporting Information). The *T*_d_ was defined as the temperature at which a 5 wt% weight loss occurred in the TGA thermogram. The *T*_d_s of ICzF and DICzF were determined to be 498 and 539 °C, respectively. The rigid polyaromatic structure of ICzF contributed to its high thermal stability, and the extended conjugation structure of DICzF further enhanced its thermal stability.

**Figure 4:**
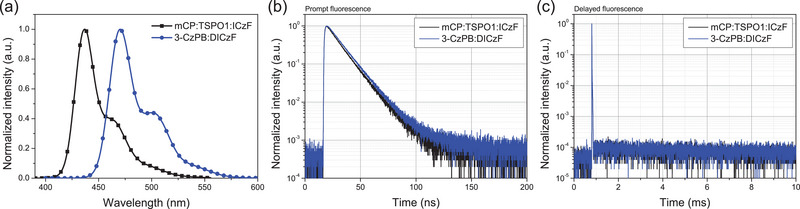
Steady state PL and TRPL analysis measured in the 3 wt% doped mCP:TSPO1:ICzF (black) and 3‐CzPB:DICzF (blue) film. a) Solid PL spectra. b) Decay curves of prompt fluorescence component. c) Decay curves of delayed fluorescence component.

PLQY and transient PL (TRPL) characterizations were also conducted to investigate the TADF nature of the ICzF and DICzF emitters. **Table** **2** summarizes the photophysical characteristics related to radiative transition of the doped films, and **Figure** **4** provides the steady‐state PL emission spectra and TRPL decay curves. A solid film with a 3 wt% emitter doped in a mCP:TSPO1 host was used for the PLQY and TRPL analysis. The PLQYs of the ICzF and DICzF emitters were 83% and 86% under nitrogen, respectively. The DICzF recorded a higher PLQY than ICzF because the additional indole unit performs as a chromophore through the MR structure. This indicates that expanded MR structure is advantageous to reach high PLQY in the MR emitters. TRPL data delivered prompt and delayed decay curves although the delayed emission was relatively slow because of rather large Δ*E*_ST_. Although the delayed emission was retarded, TADF emission was clearly observed in the two emitters from the TRPL measurement, proving that the ICzF and DICz emitters are MR–TADF emitters.

**Table 2:** Summarized photophysical characteristics of ICzF and DICzF measured in the 3 wt% doped film.

	Host	λ_PL_ ^a)^	FWHM	PLQY^b)^	*Φ*_PF_ ^c)^	*Φ*_DF_ ^d)^	*τ*_p_ ^e)^	*τ*_d_ ^f)^	*k*_ISC_ ^g)^	*k*_RISC_ ^h)^	*k*_r_^S^ ^i)^
		[nm]	[nm]	[%]	[%]	[%]	[ns]	[ms]	(10^7^ s^−1^)	(10^2^ s^−1^)	(10^7^ s^−1^)
ICzF	mCP:TSPO1	437	25	83	50	33	11.2	7.49	4.46	1.76	4.46
DICzF	3‐CzPB	471	28	86	50	36	12.1	14.42	4.13	1.00	4.13

^a)^ Value at the peak position;

^b)^ Absolute PLQY under a nitrogen atmosphere;

^c)^ Prompt fluorescence component;

^d)^ Delayed fluorescence component;

^e)^ Fitted decay time of prompt fluorescence;

^f)^ Fitted decay time of delayed fluorescence;

^g)^ Rate constant of intersystem crossing;

^h)^ Rate constant of reverse intersystem crossing;

^i)^ Rate constant of radiative singlet emission.

### 2.3 Device Characterization

**Figure 5:**
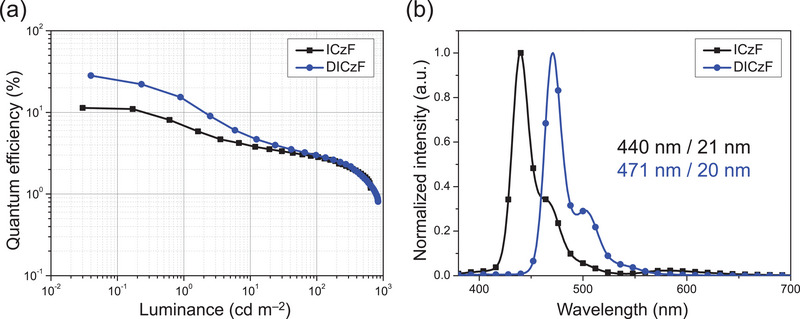
Device performance of 3 wt% emitter‐embedded blue MR–TADF devices (black: ICzF; blue: DICzF). a) External quantum efficiency–luminance curves. b) Normalized electroluminescence spectra.

The ICzF and DICzF TADF materials were evaluated as a blue emitter in the device structure harvesting the triplet excitons. The device structure of MR–TADF devices was ITO (50 nm)/PEDOT:PSS (40 nm)/TAPC (10 nm)/TCTA (5 nm)/mCP (5 nm)/EML (25 nm)/TSPO1 (25 nm)/LiF (1.5 nm)/Al (200 nm), where ITO is indium tin oxide, PEDOT:PSS is poly(3,4‐ethylenedioxythiophene), TAPC is 4,4′‐(cyclohexane‐1,1‐diyl)bis(*N*,*N*‐di‐*p*‐tolylaniline), TCTA is tris(4‐(9*H*‐carbazol‐9‐yl)phenyl)amine. The hosts of ICzF‐ and DICzF‐embedded devices were mCP:TSPO1 (50 wt%:50 wt%) and 3‐CzPB, respectively, and the doping concentration of emitter was set to 3 wt%, respectively. A mCP:TSPO1 mixed host was used in the ICzF device due to high emission energy of the emitter, while 3‐CzPB single host was used in the DICzF device. The energy level diagram of the device structures and the materials used to fabricate the devices are illustrated in Figure S22 (Supporting Information). EQE–luminance data and EL spectra of the devices are presented in **Figure** **5**. The device performance summary of blue MR‐TADF devices is provided in **Table** **3**. The current density–voltage–luminance, current efficiency–luminance, and power efficiency–luminance curves, are described in Figure S23 (Supporting Information). The maximum EQEs of the ICzF and DICzF devices were 11.3% and 28.2%, respectively. High EQEs were achieved in the DICzF device through the high PLQY of 86%, efficient RISC process, and horizontal emitting dipole orientation ratio (*θ*_EDO_) of 95% (Figure S24, Supporting Information). Although the DICzF molecule does not have any electron deficient unit, high EQE was realized by MR–TADF emission, suggesting the potential of the donor only molecular structure for highly efficient emitters. In the case of ICzF, the EQE was relatively low due to low PLQY and low *θ*_EDO_ ratio of 83%. In particular, high emission energy of the ICzF emitter made it difficult to fully harvest the excitons of it in the emitting layer. EL spectra of the ICzF and DICzF devices provided a peak wavelength of EL emission (*λ*_EL_)/FWHM of 440 nm/21 nm and 471 nm/20 nm, respectively. Both emitters achieved narrow EL emission by the MR structure although the *λ*_EL_ was different depending on the degree of expansion of the conjugated MR structure. The second vibrational peak was suppressed in the DICzF emitter due to the more rigid backbone structure.

**Table 3:** Summarized device performance of ICzF and DICzF‐embedded blue MR–TADF devices.

	Host	*V*_d_ ^a)^	λ_EL_ ^b)^	FWHM^a)^	CIE (x, y)^a)^	EQE_Max_	CE_Max_	Blue index^c)^	PE_Max_
		[V]	[nm]	[nm]		[%]	(cd A^−1^)	(cd A^−1^)	(lm W^−1^)
ICzF	mCP:TSPO1	5.8	440	21	(0.169, 0.054)	11.3	5.0	92.6	4.6
DICzF	3‐CzPB	4.8	471	20	(0.120, 0.184)	28.2	37.6	204.3	39.3

^a)^ Value at 100 cd m^−2^;

^b)^ Value at peak position;

^c)^ Defined as CE_Max_/CIE_y_.

## 3 Conclusion

In this study, a novel molecular design approach was developed for blue MR‐TADF emitters, ICzF and DICzF, which exhibit a narrow emission spectrum and high efficiency. These blue MR‐TADF emitters were designed utilizing ICz and DICz backbone structures in combination with a spiro[3,2,1‐*de*]acridine moiety. The backbone structures secured the excellent rigidity of the polyaromatic framework, leading to small FWHM_PL_ values of 16 and 15 nm for ICzF and DICzF, respectively. In addition, the peripheral spirofluorene groups in ICzF and DICzF provided substantial molecular bulkiness, effectively implementing an intermolecular blocking function. Notably, these emitters exhibited a small Δ*E*_ST_ of ≈0.33 eV despite the absence of any electron accepting unit, marking the first instance of TADF activity in purely electron donating structures. The additional indole expansion in DICzF further enhanced its performance, achieving a high *θ*_EDO_ of 95%. As a result, the DICzF‐embedded blue MR‐TADF device demonstrated a high EQE of 28.2% and a small FWHM_EL_ of 20 nm, thereby proving the superiority of the proposed molecular design strategy. These results present another approach for the future development of highly efficient, narrow‐bandwidth blue emitters.

## Conflict of Interest

The authors declare no conflict of interest.

## Supporting information



Supporting Information

## Data Availability

The data that support the findings of this study are available from the corresponding author upon reasonable request.
