# Supplementary material for: Diindolocarbazole as a Core Structure for Narrow‐Emitting and Highly Efficient Blue Organic Light‐Emitting Diodes
Source: Adv Sci (Weinh). 2025 Jun 10;12(32):e04625. doi: 10.1002/advs.202504625 (PMC12407347; doi:10.1002/advs.202504625)
Supplement: Supplementary file 1 — Supporting Information [file ADVS-12-e04625-s001.docx]

**Supporting Information**

Diindolocarbazole as a Core Structure for Narrow-Emitting and Highly Efficient Blue Organic Light-Emitting Diodes

Jihoon Kang^1†^, Junyoung Moon^1†^, Soon Ok Jeon^2^, Unhyeok Jo^1^, Seungwon Han^3^, Sangmo Kim^2*^, Jun Yeob Lee^1,3,4*^

^1^School of Chemical Engineering, Sungkyunkwan University

2066, Seobu-ro, Jangan-gu, Suwon, Gyeonggi, 16419, Republic of Korea

E-mail: [leej17@skku.edu](mailto:leej17@skku.edu)

^2^Samsung Advanced Institute of Technology, Samsung Electronics Co., Ltd.,

130 Samsung-ro, Yeongtong-gu, Suwon, Gyeonggi, 16678, Republic of Korea

E-mail: [sang1770.kim@samsung.com](mailto:sang1770.kim@samsung.com)

^3^Department of Display Convergence Engineering, Sungkyunkwan University

2066, Seobu-ro, Jangan-gu, Suwon, Gyeonggi, 16419, Republic of Korea

^4^SKKU Institute of Energy Science and Technology, Sungkyunkwan University

2066, Seobu-ro, Jangan-gu, Suwon, Gyeonggi, 16419, Republic of Korea

^†^ These authors contributed equally to this work

^*^ To whom correspondence should be addressed

**Supporting Information**

**1. Synthesis**

***1.1. General information***

***1.2. Synthetic procedure***

**2. Quantum chemical calculation**

**3. Material characterization**

**4. Device characterization**

**5. Supplementary figures**

**6. Supplementary tables**

**Supplementary references**

**1. Synthesis**

***1.1. General information***

All reagents, catalysts, bases, and solvents used in this paper are commercially available. The starting materials, 2,7-dibromo-9*H*-carbazole, 1,4-dibromo-2,5-diiodobenzene were purchased from companies. The starting material, 2,7-di-*tert*-butyl-10*H*-spiro[acridine-9,9'-fluorene] (SM1) was prepared according to the synthetic method reported in the literature.^[43]^

^1^H, ^13^C nuclear magnetic resonance (NMR) spectra were recorded at 500 MHz NMR instrument using chloroform-*d* (CDCl_3_), methylene chloride-*d*_2_ (CD_2_Cl_2_), and dimethyl sulfoxide-*d*_6_ (DMSO-*d*_6_) solvent. For mass spectroscopy, a low-resolution mass spectrometer (Advion, Expression) using atmospheric pressure chemical ionization (APCI) source was used. A high-resolution mass spectroscopy (HRMS) analysis was conducted using JMS-700(JEOL) instrument under high resolution-fast atom bombardment mode.

***1.2. Synthetic procedure***

Scheme 1. Synthesis scheme of ICzF and DICzF.

**4-Bromo-2,7-di-*tert*-butyl-10*H*-spiro[acridine-9,9'-fluorene] (1)**

2,7-Di-*tert*-butyl-10*H*-spiro[acridine-9,9'-fluorene] (SM1) (0.74 g, 1.70 mmol) was dissolved in *N*,*N*-dimethylformamide (DMF) (10 mL) under a nitrogen atmosphere. *N*-bromosuccinimide (NBS) (0.30 g, 1.70 mmol) was added at 0 °C, and the mixture was stirred at room temperature for 1 h. Upon completion of the reaction, the organic layer was extracted with ethyl acetate (EA) and deionized water (DW). The collected organic layer was dried over anhydrous magnesium sulfate (MgSO_4_) and concentrated under reduced pressure. The crude product was purified via column chromatography using an *n*-hexane (Hx)/methylene chloride (MC) (4:1) mobile phase. The white solid was obtained by filtration with methanol (0.74 g, yield: 83%).

^1^H NMR (500 MHz, CDCl_3_) δ 7.76 (dt, *J* = 7.5, 1.0 Hz, 2H), 7.37 – 7.29 (m, 4H), 7.19 (td, *J* = 7.5, 1.1 Hz, 2H), 7.14 – 7.09 (m, 1H), 6.88 (d, *J* = 8.4 Hz, 1H), 6.81 (d, *J* = 2.1 Hz, 1H), 6.34 (dd, *J* = 20.4, 2.3 Hz, 2H), 0.99 (s, 9H), 0.96 (s, 9H).

MS (APCI) m/z: Found 522.15 [(M + H)^+^]. Calculated for C_33_H_32_BrN: 521.17.

**2,7-Di-*tert*-butyl-4-(4,4,5,5-tetramethyl-1,3,2-dioxaborolan-2-yl)-10*H*-spiro[acridine-9,9'-fluorene] (2)**

Intermediate 1 (0.73 g, 1.40 mmol), bis(pinacolato)diboron (0.54 g, 2.10 mmol), [1,1′-bis(diphenylphosphino)ferrocene]dichloropalladium(II) (Pd(dppf)Cl_2_) (0.03 g, 0.04 mmol), and potassium acetate (KOAc) (0.42 g, 4.28 mmol) were dissolved in 1,4-dioxane (15 mL) under an N_2_ atmosphere. The mixture was refluxed overnight. Upon completion of the reaction, the organic layer was extracted with EA and DW. The collected organic layer was dried over anhydrous MgSO_4_ and concentrated under reduced pressure. The crude product was purified via column chromatography using an Hx/MC (2:1) mobile phase. The white solid was obtained by filtration with methanol (0.64 g, yield: 79%).

^1^H NMR (500 MHz, CDCl_3_) δ 7.75 (d, *J* = 7.5 Hz, 2H), 7.54 (d, *J* = 2.4 Hz, 1H), 7.35 (dt, *J* = 7.6, 0.9 Hz, 2H), 7.31 (td, *J* = 7.5, 1.1 Hz, 2H), 7.16 (td, *J* = 7.5, 1.2 Hz, 2H), 7.07 (d, *J* = 8.2 Hz, 1H), 6.76 (s, 1H), 6.47 (s, 1H), 6.34 (s, 1H), 1.41 (s, 12H), 0.98 (s, 18H).

MS (APCI) m/z: Found 570.39 [(M + H)^+^]. Calculated for C_39_H_44_BNO_2_: 569.35.

**4,4''-(2,5-Dibromo-1,4-phenylene)bis(2,7-di-*tert*-butyl-10*H*-spiro[acridine-9,9'-fluorene]) (3)**

1,4-Dibromo-2,5-diiodobenzene (0.45 g, 0.92 mmol), intermediate 2 (1.07 g, 1.90 mmol), tetrakis(triphenylphosphine)palladium(0) (Pd(PPh_3_)_4_, 0.053 g, 0.05 mmol), and potassium carbonate (K_2_CO_3_) (0.56 g, 4.05 mmol) were dissolved in toluene (5 mL), ethanol (EtOH) (3 mL), and DW (2 mL) under an N_2_ atmosphere. The mixture was refluxed overnight. Upon completion of the reaction, the organic layer was extracted with EA and DW. The collected organic layer was dried over MgSO_4_ and concentrated under reduced pressure. The crude product was purified via column chromatography using an Hx/MC (3:1) mobile phase. The white solid was obtained by filtration with methanol (0.53 g, yield: 53%).

^1^H NMR (500 MHz, CDCl_3_) δ 7.91 (s, 2H), 7.80 (dd, *J* = 10.1, 7.5 Hz, 6H), 7.49 (d, *J* = 7.5 Hz, 2H), 7.44 (d, *J* = 7.6 Hz, 2H), 7.42 – 7.38 (m, 2H), 7.38 – 7.32 (m, 2H), 7.22 (td, *J* = 7.7, 1.4 Hz, 2H), 7.12 – 7.00 (m, 4H), 6.76 (d, *J* = 8.4 Hz, 2H), 6.52 (d, *J* = 2.2 Hz, 2H), 6.41 (d, *J* = 2.2 Hz, 2H), 6.05 (s, 2H), 1.08 (s, 18H), 1.00 (s, 18H).

MS (APCI) m/z: Found 1117.42 [(M + H)^+^]. Calculated for C_72_H_66_Br_2_N_2_: 1116.36

**2,7-Dibromo-3,6-dichloro-9*H*-carbazole (4)**

2,7-Dibromo-9*H*-carbazole (SM2) (10.00 g, 30.77 mmol), *N*-chlorosuccinimide (NCS) (8.61 g, 64.48 mmol), and DMF (100 mL) were added to a 2-neck round-bottom flask. The mixture was heated to 60 °C under a N_2_ atmosphere for 6 h. Upon completion of the reaction, the mixture was quenched with a 2 M aqueous sodium thiosulfate solution and stirred for 30 minutes. The reaction mixture was then extracted with MC and DW. The collected organic phase was dried over anhydrous MgSO_4_ and concentrated under reduced pressure using a rotary evaporator. The crude product was purified via column chromatography using a Hx/tetrahydrofuran (THF) (4:1) mobile phase, followed by reprecipitation with Hx/THF. The product was obtained as a white solid (7.42 g, yield: 61%).

^1^H NMR (500 MHz, DMSO-*d*_6_) δ 11.64 (s, 1H), 8.50 (s, 2H), 7.89 (s, 2H).

MS (APCI) m/z: Found 391.88 [(M + H)^+^]. Calculated for C_12_H_5_Br_2_Cl_2_N: 390.82

**2,7-Dibromo-3,6-dichloro-9-phenyl-9*H*-carbazole (5)**

Intermediate 4 (3.00 g, 7.62 mmol), iodobenzene (2.33 g, 11.42 mmol), copper(I) iodide (CuI) (0.29 g, 1.52 mmol), 1,10-phenanthroline (0.27 g, 1.52 mmol), K_2_CO_3_ (3.16 g, 22.85 mmol), and DMF (30 mL) were added to a 2-neck round-bottom flask. The mixture was refluxed under an N_2_ atmosphere for 12 h. Upon completion of the reaction, the mixture was cooled to room temperature and diluted with MC. The mixture was passed through a short celite/silica-packed filter and concentrated under reduced pressure using a rotary evaporator. The crude product was purified via column chromatography using a mobile phase of Hx/MC (1:4), followed by reprecipitation from Hx/MC. The product was obtained as a white solid (0.60 g, yield: 17%).

^1^H NMR (500 MHz, CDCl_3_) δ 8.11 (s, 2H), 7.67 – 7.61 (m, 2H), 7.57 (s, 2H), 7.56 – 7.52 (m, 1H), 7.47 – 7.44 (m, 2H).

MS (APCI) m/z: Found 467.86 [(M + H)^+^]. Calculated for C_18_H_9_Br_2_Cl_2_N: 466.85

**4,4''-(3,6-Dichloro-9-phenyl-9*H*-carbazole-2,7-diyl)bis(2,7-di-*tert*-butyl-10*H*-spiro[acridine-9,9'-fluorene]) (6)**

Intermediate 5 (0.30 g, 0.64 mmol), intermediate 2 (0.91 g, 1.60 mmol), Pd(PPh_3_)_4_ (0.07 g, 0.06 mmol), SPhos (0.05 g, 0.13 mmol), tripotassium phosphate (K_3_PO_4_) (4.24 g, 20.00 mmol), 1,4-dioxane (10 mL), and DW (5 mL) were added to a 2-neck round-bottom flask. The mixture was refluxed under a nitrogen atmosphere for 1 hour. The purification of intermediate 6 followed the procedure used for intermediate 3. The product was obtained as a white solid (0.71 g, yield: 93%).

^1^H NMR (500 MHz, CD_2_Cl_2_) δ 8.43 (s, 2H), 7.81 (ddd, *J* = 7.7, 3.1, 2.1 Hz, 4H), 7.64 (dd, *J* = 8.5, 1.3 Hz, 2H), 7.61 – 7.56 (m, 2H), 7.54 (s, 2H), 7.50 – 7.42 (m, 3H), 7.42 – 7.33 (m, 4H), 7.35 – 7.32 (m, 2H), 7.25 (tt, *J* = 7.4, 1.0 Hz, 2H), 7.21 (tt, *J* = 7.4, 1.4 Hz, 2H), 7.05 (dp, *J* = 6.6, 2.2 Hz, 4H), 6.64 (dd, *J* = 8.4, 2.6 Hz, 2H), 6.45 (d, *J* = 2.2 Hz, 2H), 6.40 – 6.31 (m, 2H), 6.13 (d, *J* = 5.8 Hz, 2H), 1.00 (s, 18H), 0.96 (s, 9H), 0.96 (s, 9H).

MS (APCI) m/z: Found 1194.66 [(M + H)^+^]. Calculated for C_84_H_73_Cl_2_N_3_: 1193.52

**ICzF**

Intermediate 3 (0.53 g, 0.47 mmol), CuI (0.04 g, 0.19 mmol), 1,10-phenanthroline (0.07 g, 0.38 mmol), and K_3_PO_4_ (0.40 g, 1.90 mmol) were dissolved in DMF (4 mL) under an N_2_ atmosphere. The mixture was refluxed overnight. Upon completion of the reaction, the organic layer was extracted with MC and DW. The collected organic layer was dried over MgSO_4_ and concentrated under reduced pressure. The crude product was purified via column chromatography using an Hx/MC (3:1) mobile phase. The final product was obtained as a yellow solid by filtration with acetone (0.27 g, yield: 54%).

^1^H NMR (500 MHz, CD_2_Cl_2_) δ 8.96 (s), 8.39 (d, *J* = 8.6 Hz), 8.14 (d, *J* = 1.6 Hz), 7.97 – 7.82 (m), 7.47 (dd, *J* = 8.6, 2.3 Hz), 7.40 (ddd, *J* = 7.7, 6.9, 1.6 Hz), 7.26 – 7.15 (m), 6.63 (dd, *J* = 9.9, 2.0 Hz), 1.23 (s), 1.06 (s).

^13^C NMR (126 MHz, CD_2_Cl_2_) δ 154.92, 145.83, 139.73, 136.71, 134.64, 128.16, 127.78, 126.41, 125.53, 125.36, 124.80, 122.52, 120.57, 120.18, 114.67, 113.50, 105.36, 34.89, 34.06, 31.64, 30.84.

MS (HRMS) m/z: Found 956.5079 [(M)^+^]. Calculated for C_72_H_64_N_2_: 956.5070.

**DICzF**

Intermediate 6 (0.53 g, 0.44 mmol), CuI (0.08 g, 0.44 mmol), 1,10-phenanthroline (0.08 g, 0.44 mmol), K_3_PO_4_ (0.36 g, 1.67 mmol), and DMF (8 mL) were added to a reaction vial. The mixture was heated to 100 °C for 24 h. The reaction was quenched with DW and stirred for 30 minutes, followed by filtration with DW. After drying in a vacuum oven, the crude product was purified via column chromatography using an Hx/MC (2:1) mobile phase, followed by reprecipitation with methanol. The final product was obtained as a yellow solid (0.44 g, yield: 88%).

^1^H NMR (500 MHz, CD_2_Cl_2_) δ 9.19 (d, *J* = 0.8 Hz, 2H), 8.55 (d, *J* = 8.6 Hz, 2H), 8.23 (d, *J* = 0.7 Hz, 2H), 7.97 (d, *J* = 1.7 Hz, 2H), 7.92 (dt, *J* = 7.8, 1.2 Hz, 2H), 7.89 (dt, *J* = 7.7, 0.9 Hz, 4H), 7.81 (dd, *J* = 8.3, 7.6 Hz, 2H), 7.64 – 7.59 (m, 1H), 7.56 (dd, *J* = 8.6, 2.4 Hz, 2H), 7.41 (td, *J* = 7.5, 1.2 Hz, 4H), 7.26 (ddd, *J* = 7.6, 1.2, 0.7 Hz, 4H), 7.21 (td, *J* = 7.4, 1.1 Hz, 4H), 6.69 (d, *J* = 2.3 Hz, 2H), 6.61 (d, *J* = 1.6 Hz, 2H), 1.19 (s, 18H), 1.10 (s, 18H).

^13^C NMR (126 MHz, CD_2_Cl_2_) δ 154.89, 145.63 (d, *J* = 11.5 Hz), 139.77, 138.57, 138.35, 136.94, 135.38, 134.63, 130.42, 129.02, 128.17, 127.81, 127.61, 127.50, 126.91, 125.50, 125.41, 124.85, 124.16, 123.57, 122.48, 120.47, 114.82, 113.50, 104.43, 100.99, 57.75, 34.83, 34.12, 31.62, 30.92.

MS (HRMS) m/z: Found 1122.5743 [(M + H)^+^]. Calculated for C_84_H_71_N_3_: 1121.5648.


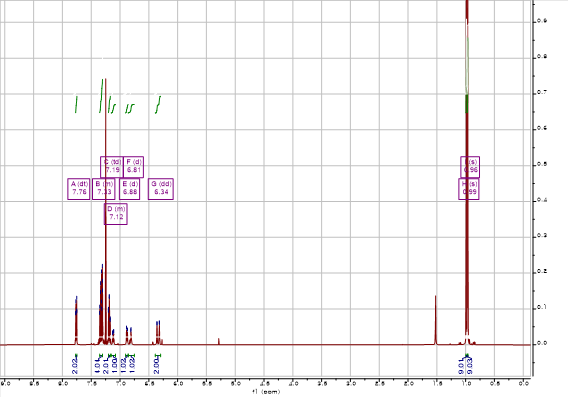


Figure S1. ^1^H NMR spectrum of intermediate 1.


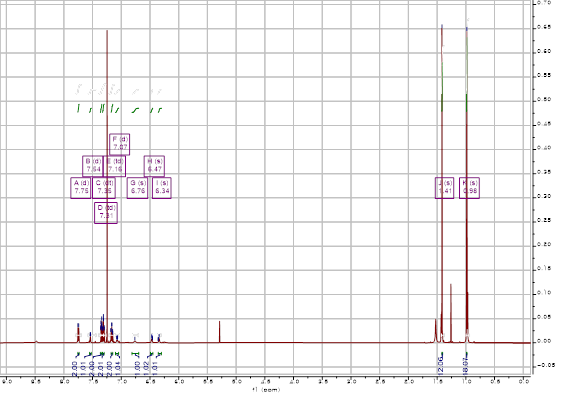


Figure S2. ^1^H NMR spectrum of intermediate 2.


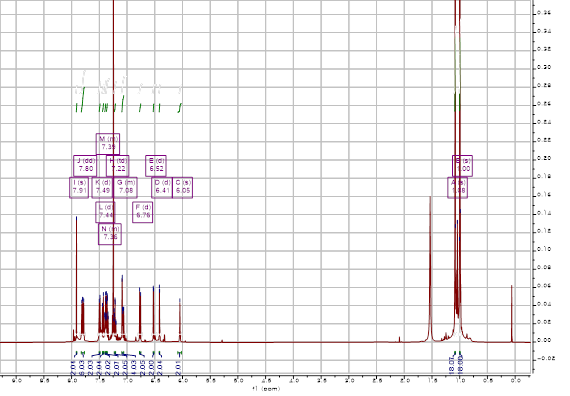


Figure S3. ^1^H NMR spectrum of intermediate 3.


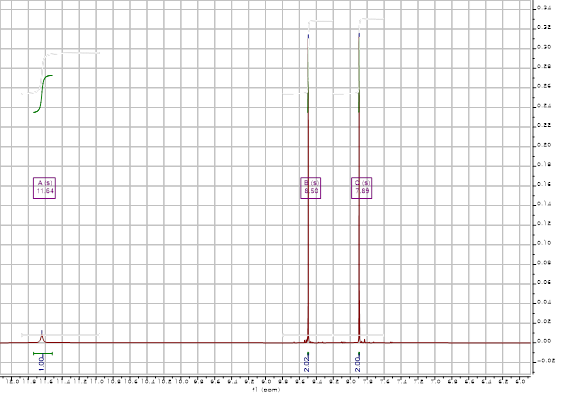


Figure S4. ^1^H NMR spectrum of intermediate 4.


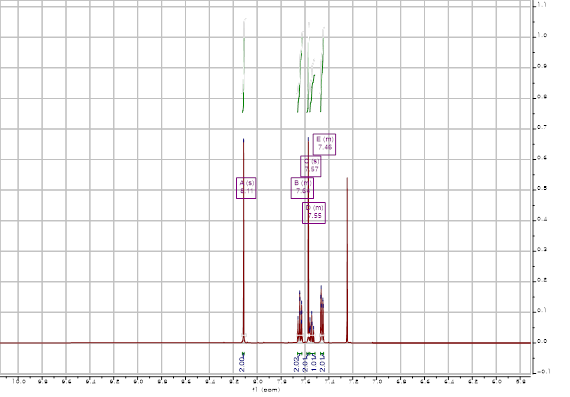


Figure S5. ^1^H NMR spectrum of intermediate 5.


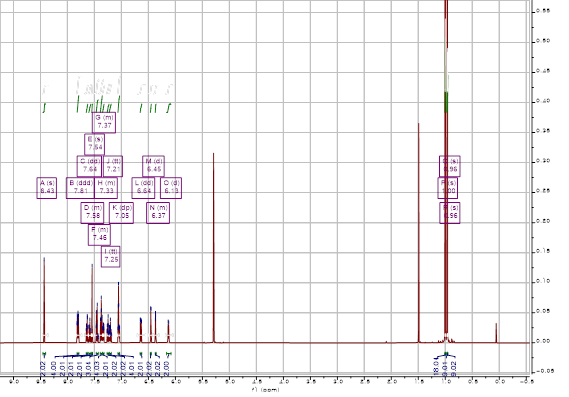


Figure S6. ^1^H NMR spectrum of intermediate 6.


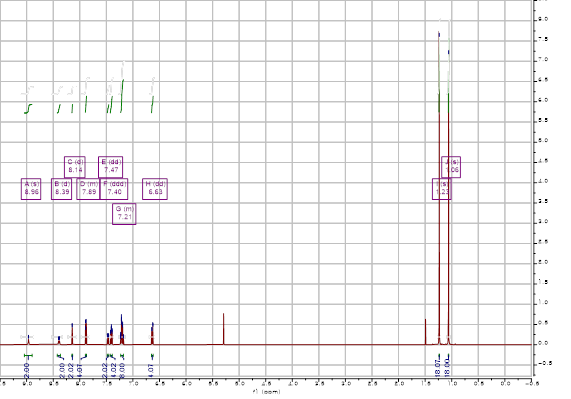


Figure S7. ^1^H NMR spectrum of ICzF.


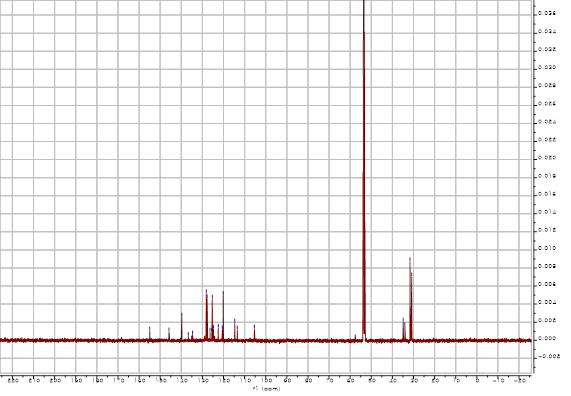


Figure S8. ^13^C NMR spectrum of ICzF.


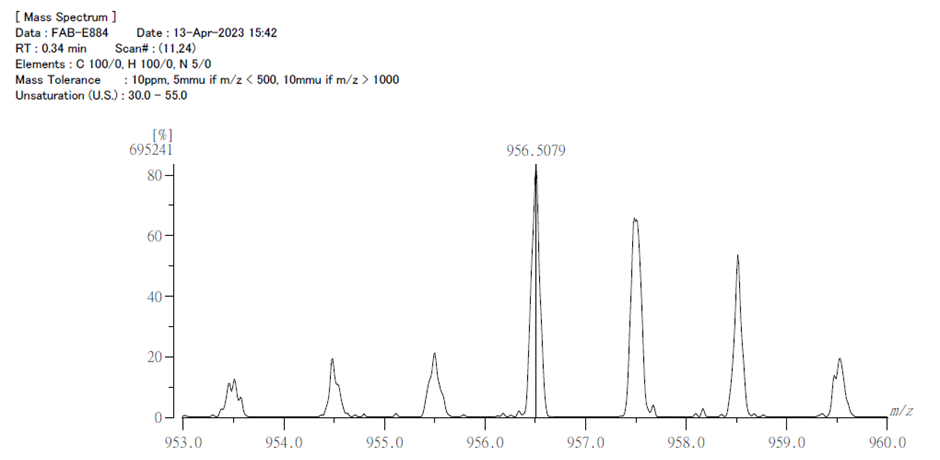


Figure S9. HRMS spectrum of ICzF.


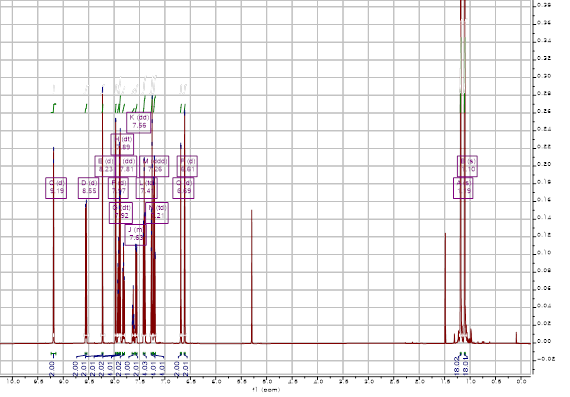


Figure S10. ^1^H NMR spectrum of DICzF.


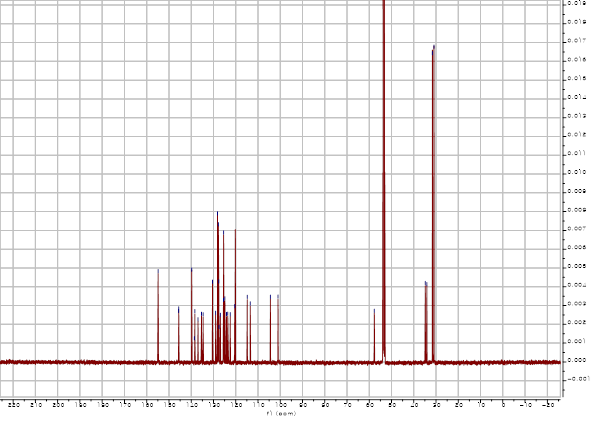


Figure S11. ^13^C NMR spectrum of DICzF.


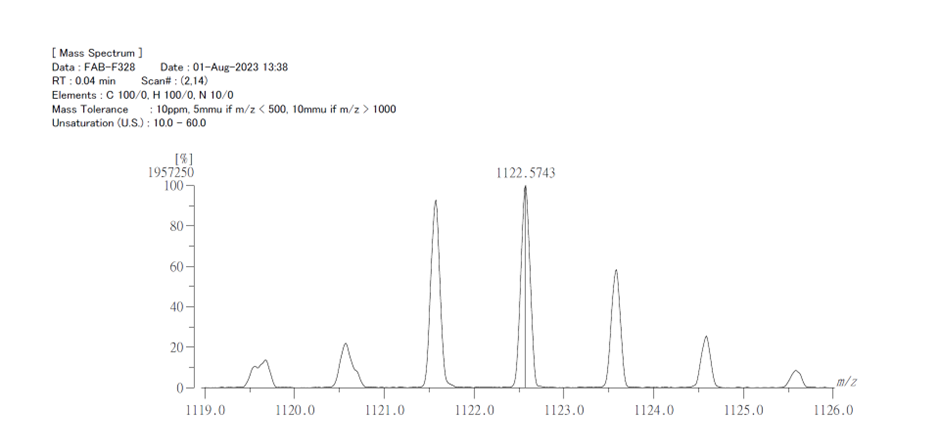


Figure S12. HRMS spectrum of DICzF.

**2. Quantum chemical calculation**

The density functional theory (DFT) calculation was performed using Gaussian 16W software.^[44]^ The optimization of ground state (S_0_), 1^st^ singlet excited state (S_1_), and 1^st^ triplet excited state (T_1_) geometry were calculated by using the hybrid version of the Becke’s three parameter exchange functional with the Lee-Yang-Parr correlation functional (B3LYP)^[45-48]^ at 6-31g(*d*) level. The excited state properties of molecules were predicted by time dependent-DFT (TD-DFT) calculation at optimized S_0_ geometry to obtain vertical excitation energy, and optimization of S_1_ and T_1_ geometry to obtain vertical emission energy and the natural transition orbital (NTO) distributions. The highest occupied NTO (HONTO) and the lowest unoccupied NTO (LUNTO) distributions of S_1_ state and triplet excited states (T_1_–T_4_ state) were calculated at optimized S_1_ and T_1_ geometry, respectively. The molecular cubic of molecules was determined at optimized S_0_ geometry using Multiwfn software.^[55]^

**3. Material characterization**

Ultraviolet–visible (UV–Vis) absorption spectra were recorded using a UV–Vis spectrophotometer (JASCO, V-730). Fluorescence and phosphorescence spectra were measured using a fluorescence spectrometer (PerkinElmer, LS-55). The samples were dissolved in THF and toluene at a concentration of 10^–5^ M. Absorption spectra were recorded at room temperature, fluorescence spectra were measured at both room temperature and 77 K, and phosphorescence spectra were obtained at 77 K with a 1 ms delay.

Radiative transition analysis of the solid thin film was performed using absolute PL quantum yield (PLQY) and transient photoluminescence (PL) analysis. PLQY was measured using a Quantaurus-QY system (Hamamatsu, C11347-11) and transient PL decay measurements were performed using a Quantaurus-Tau system (Hamamatsu, C11367-31) under nitrogen condition. The 3 wt% emitter-doped films were prepared using 3,3′-di(9*H*-carbazol-9-yl)-1,1′-biphenyl (mCP):diphenyl(4-(triphenylsilyl)phenyl)phosphine oxide (TSPO1) in a ratio of 50 wt%:50 wt% for ICzF, and 2,6-bis(3-(9*H*-carbazol-9-yl)phenoxy)benzonitrile (3-CzPB) for DICzF, respectively. The thickness of the doped thin films was set to 40 nm.

The rate constants were calculated according to the following equations^[S1]^:


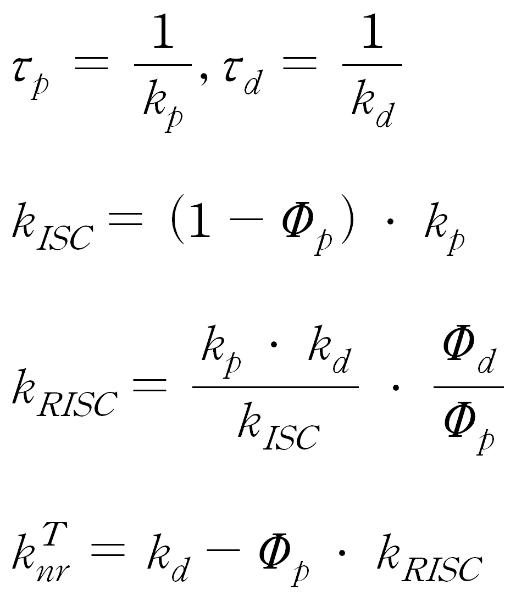


In these equations, *τ*ₚ and *k*ₚ denote the decay time and rate constant of the prompt fluorescence, respectively, while *τ*_d_ and *k*_d_ denote the decay time and rate constant of the delayed fluorescence. The *k*_ISC_ and *k*_RISC_ denote the intersystem crossing and reverse intersystem crossing rate constants, and *k*^T^_nr_ denote the triplet non-radiative decay rate constant. The *Φ*ₚ denote PLQY of prompt fluorescence component and the *Φ*_d_ denote PLQY of delayed fluorescence component, respectively.

For angle‐dependent PL measurements, an encapsulated 3 wt% doped film was affixed to a half cylinder placed on a rotating stage using refractive index‐matching oil. A 325 nm He/Cd laser served as the excitation source, and the *p*‐polarized PL emission at various angles was recorded using a MAYA 2000 Pro spectrometer (Ocean Optics Inc.).

Thermal decomposition temperature (*T*_d_) was determined using a thermogravimetric analyzer (TGA) (Hitachi High-Tech, TG/DTA7300) under a nitrogen atmosphere. The samples were heated from room temperature to 600 °C at a rate of 10 °C/min, and the temperature at which 5% mass loss occurred was taken as *T*_d_.

Cyclic voltammetry (CV) measurements to determine the energy level of the highest occupied molecular orbitals (*E*_HOMO_) were carried out using an Iviumstat (Ivium Tech.). Pt wires were employed as both the working electrode and counter electrode, and an Ag/AgCl (saturated in KCl) electrode employed as the reference electrode. Tetrabutylammonium perchlorate dissolved in methylene chloride was used as the supporting electrolyte at a concentration of 10^–3^ M, while the sample was present at a concentration of 0.1 M. The *E*_HOMO_ was determined from the onset potential of the measured oxidation curve. An optical bandgap (*E*_opt_) was obtained from the UV–Vis absorption spectra, and the energy level of the lowest unoccupied molecular orbitals (*E*_LUMO_) was then determined the following equation: *E*_LUMO_ = *E*_HOMO_ + *E*_opt_.

**4. Device characterization**

A transparent glass substrate bearing an indium tin oxide (ITO) anode was used as the substrate for fabricating the blue OLED. Prior to the deposition process, the ITO substrate underwent sequential cleaning in acetone, chloroform, isopropyl alcohol, and DW, followed by drying and oxygen plasma treatment. A 40 nm film of poly(3,4-ethylenedioxythiophene) polystyrene sulfonate (PEDOT:PSS) was then spin-coated at 3,200 rpm for 30 s onto the cleaned substrate and subsequently annealed at 150 °C for 15 min. Under a high vacuum of approximately 10^–7^ Torr, the organic layers and an aluminium cathode were deposited via thermal evaporation, and a glass lid was used to encapsulate the device. The current density–voltage–luminance characteristics of the devices were evaluated using a spectroradiometer (Konica Minolta, CS-2000) coupled with a source meter (Keithley 2400). The efficiency parameters including external quantum efficiency (EQE), current efficiency (CE) and power efficiency (PE) were calculated assuming Lambertian emission.

**5. Supplementary figures**


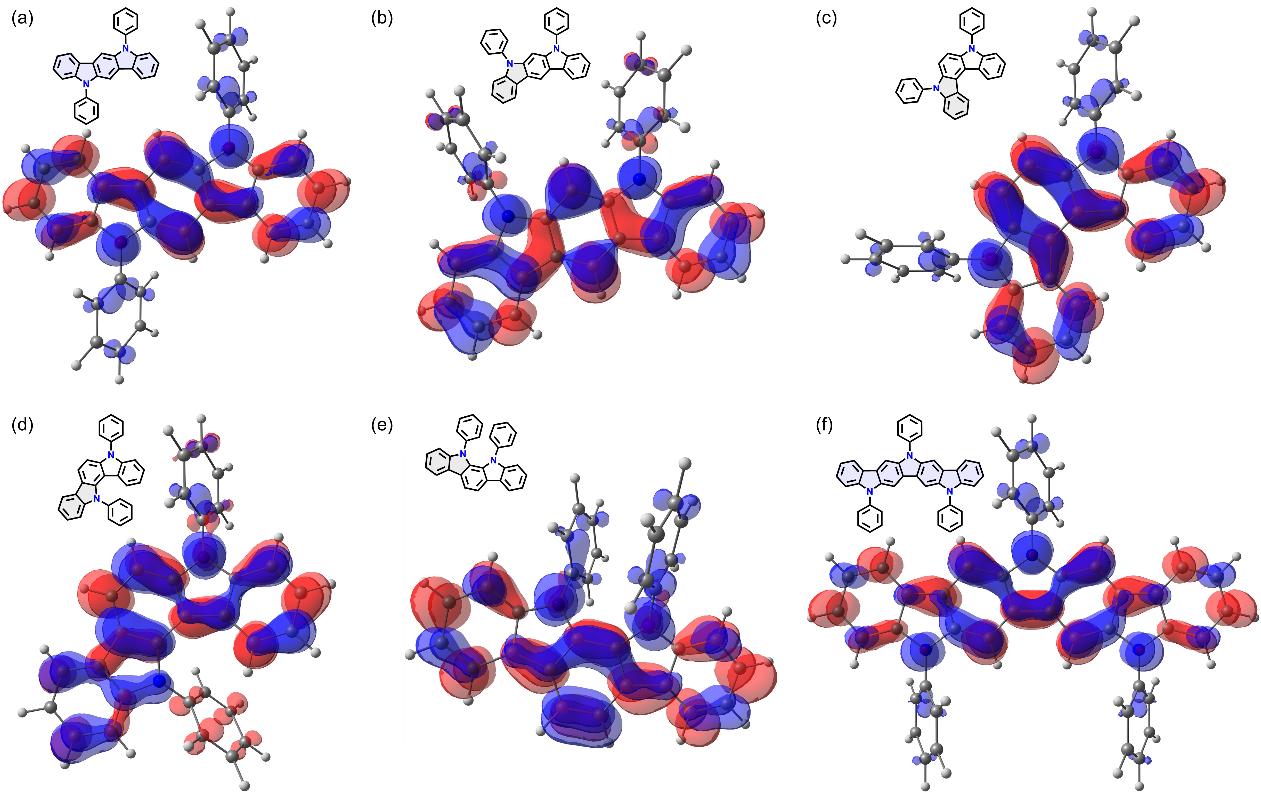


Figure S13. Frontier molecular orbital distribution of (di)indolocarbazole backbone structures. (a) ICz; (b) indolo[2,3-*b*]carbazole; (c) indolo[2,3-*c*]carbazole; (d) indolo[3,2-*a*]carbazole; (e) indolo[2,3-*a*]carbazole and (f) DICz (blue filled region represents HOMO; red filled region represents LUMO distribution).

**
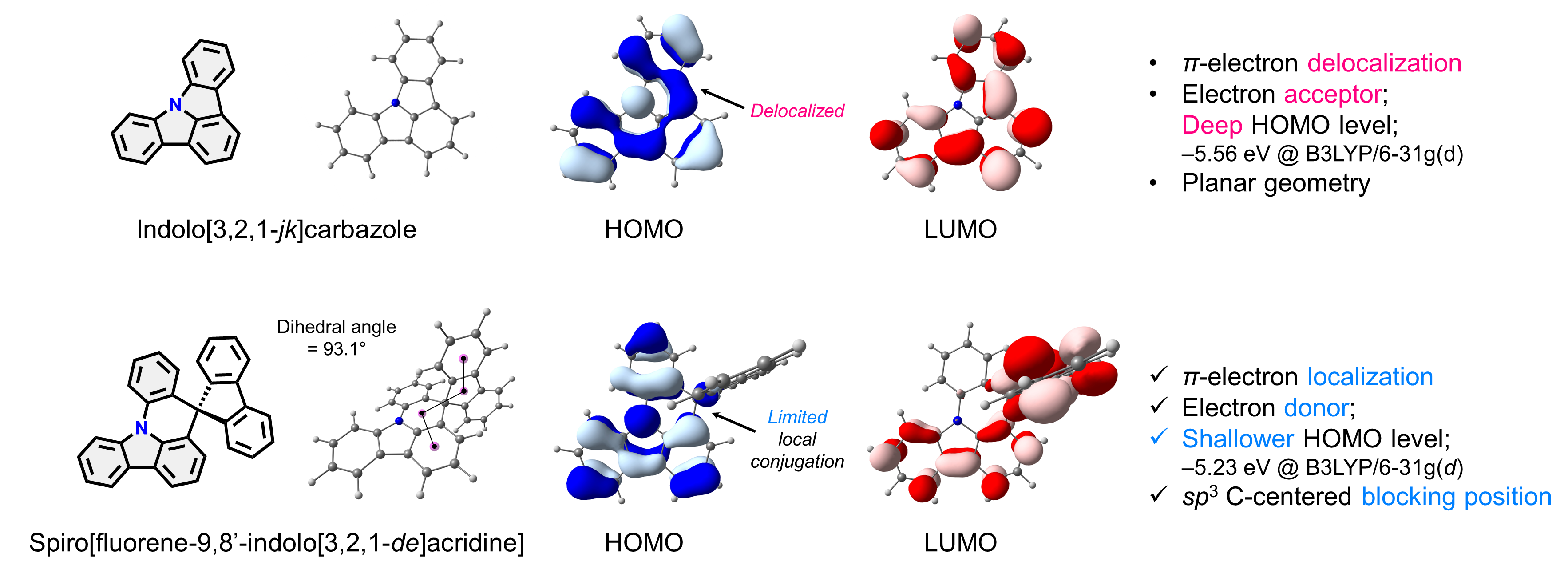
**

Figure S14. Basic specifications of indolo[3,2,1-*jk*]carbazole and spiro[fluorene-9,8’-indolo[3,2,1-*de*]acridine]. Chemical structure, optimized S_0_ geometry, HOMO (blue filled region) and LUMO (red filled region) distributions are illustrated.


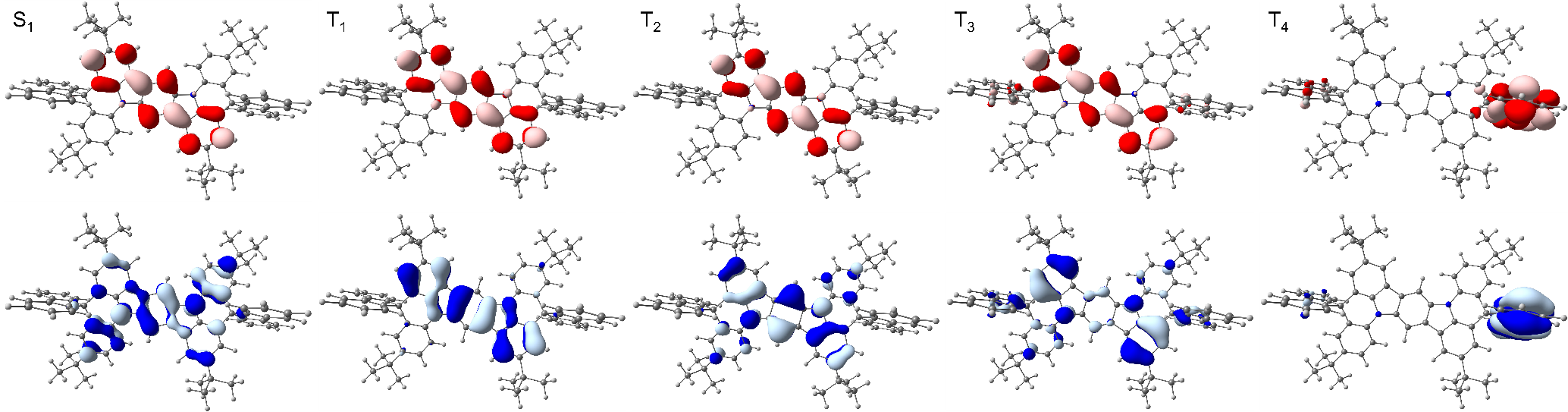


Figure S15. NTO distributions of ICzF corresponding to S_1_, T_1_–T_4_ excited state calculated at optimized S_1_ and T_1_ geometry, respectively (blue filled region represents HONTO; red filled region represents LUNTO distribution).


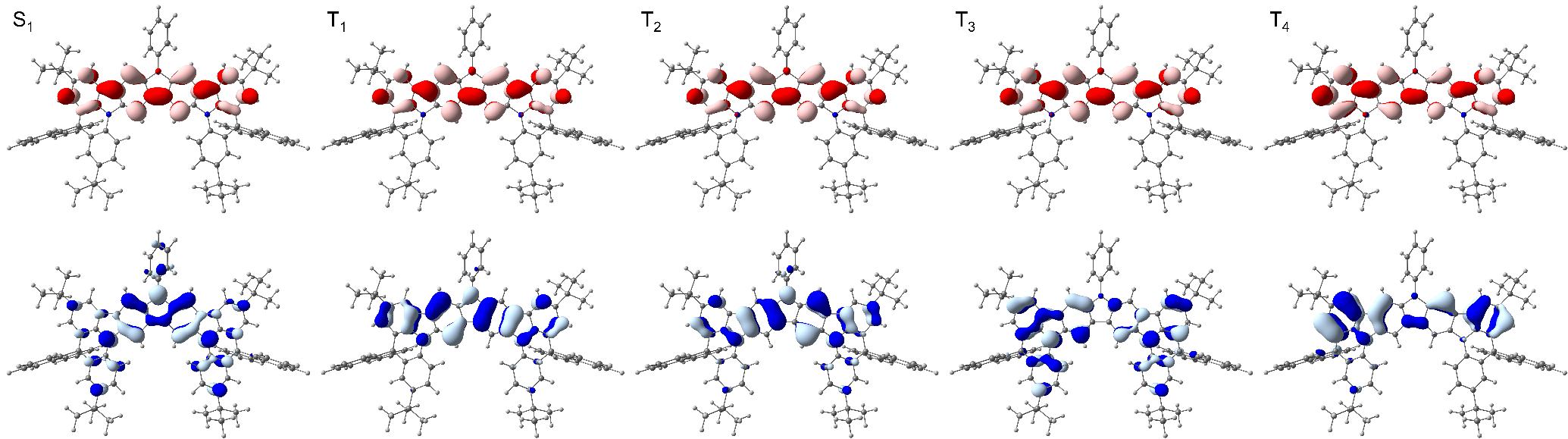


Figure S16. NTO distributions of DICzF corresponding to S_1_, T_1_–T_4_ excited state calculated at optimized S_1_ and T_1_ geometry, respectively (blue filled region represents HONTO; red filled region represents LUNTO distribution).


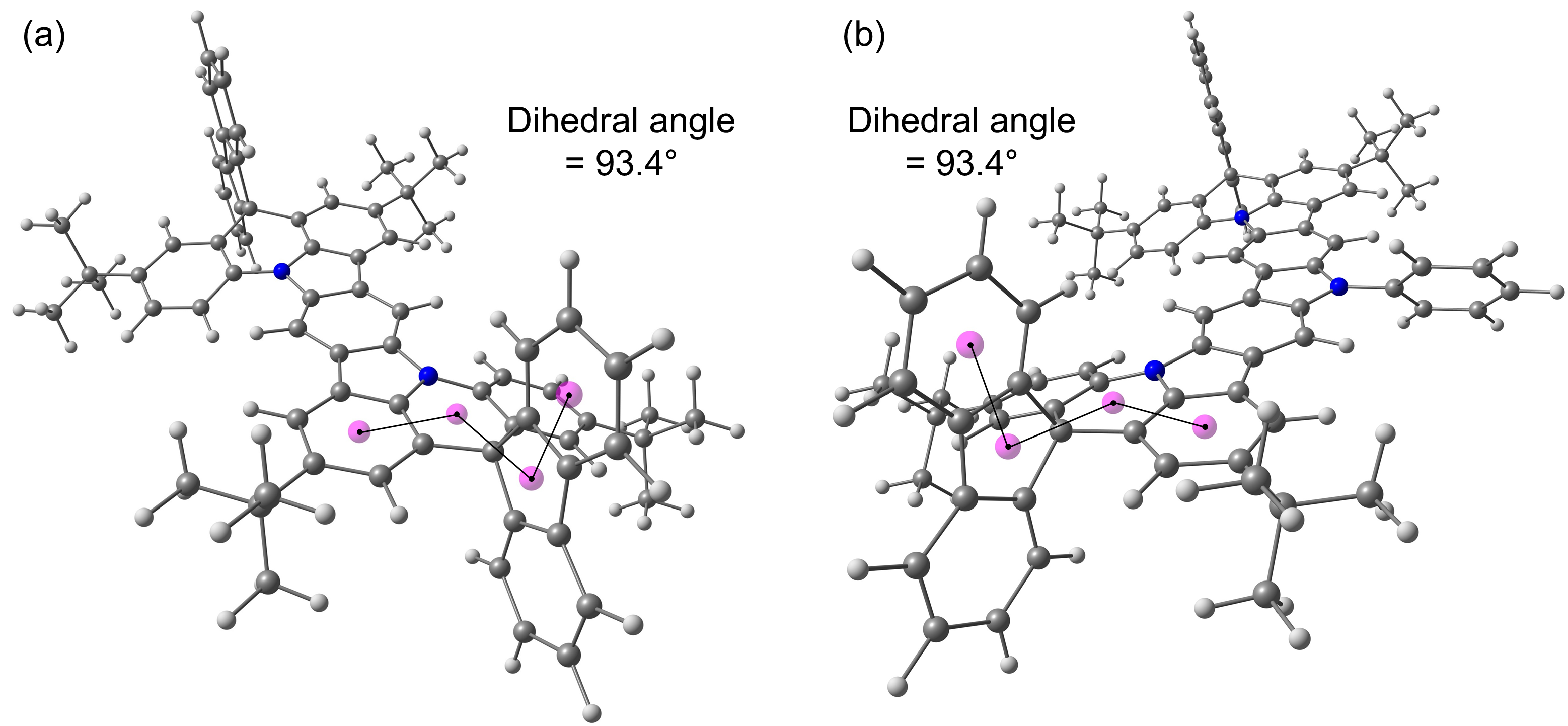


Figure S17. Dihedral angle between spirofluorene group and (di)indolocarbazole backbone of (a) ICzF and (b) DICzF.


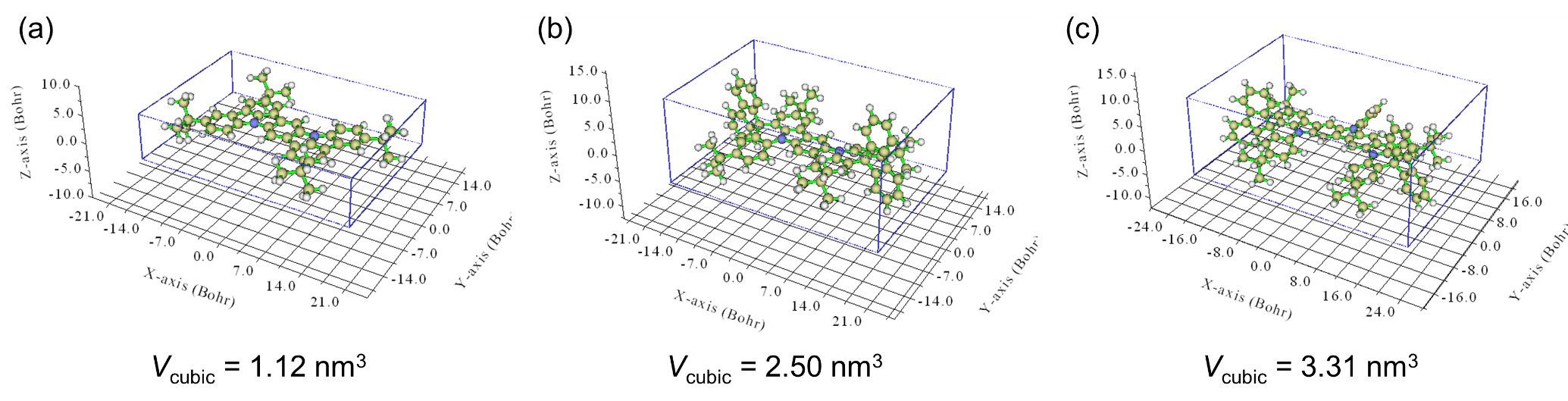


Figure S18. The molecular cubic specification and its molecular cubic volume (*V*_cubic_) of (a) tBisICz, (b) ICzF and (c) DICzF calculated at optimized S_0_ geometry.

Figure S19. Steady-state PL spectra of ICzF (black) and DICzF (blue) in diluted toluene solution at room temperature.

Figure S20. Oxidation curves of ICzF (black) and DICzF (blue) measured by cyclic voltammetry analysis.

Figure S21. TGA thermogram of ICzF (black) and DICzF (blue).


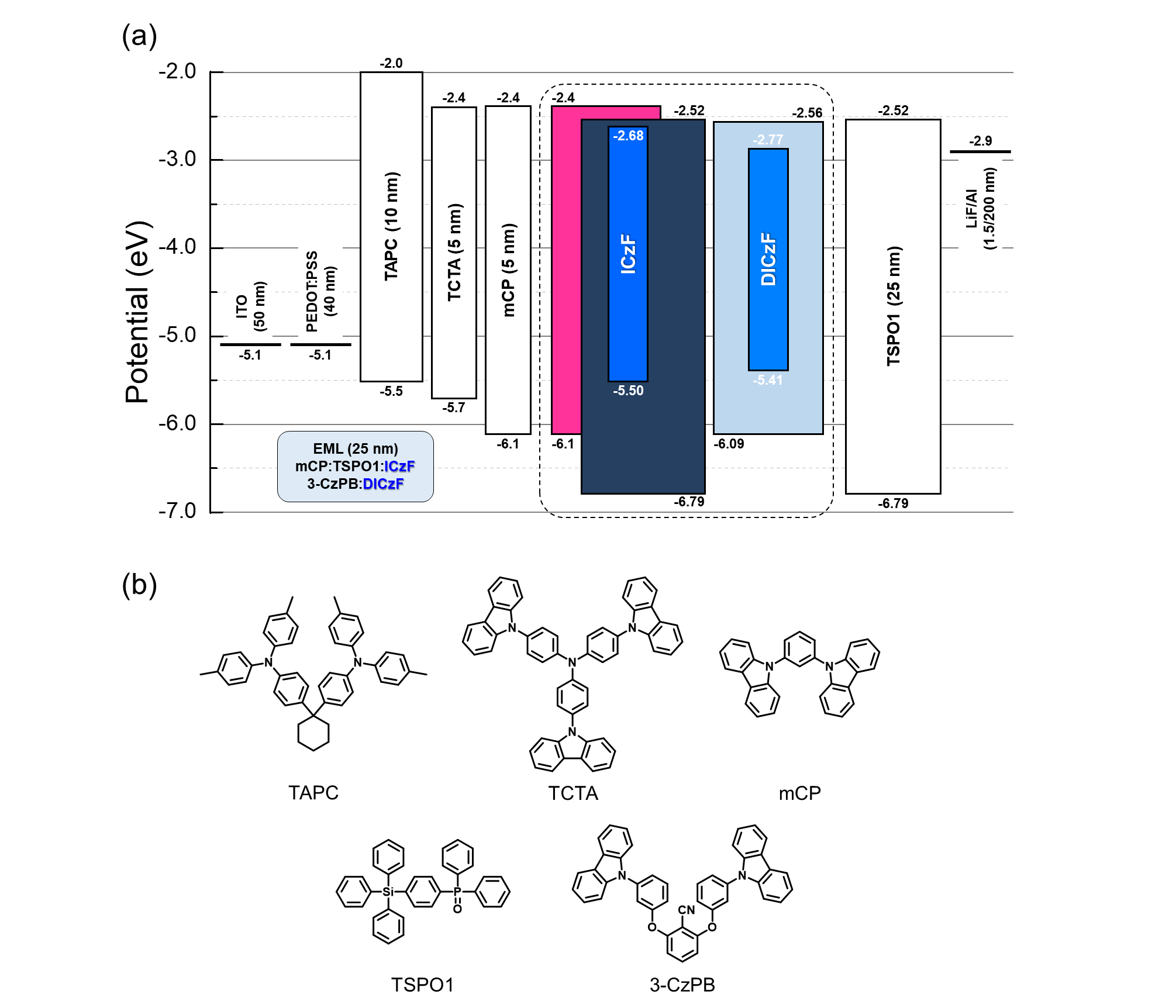


Figure S22. (a) Energy level diagram of the blue MR–TADF devices. (b) Chemical structure of materials used to fabricate the devices.


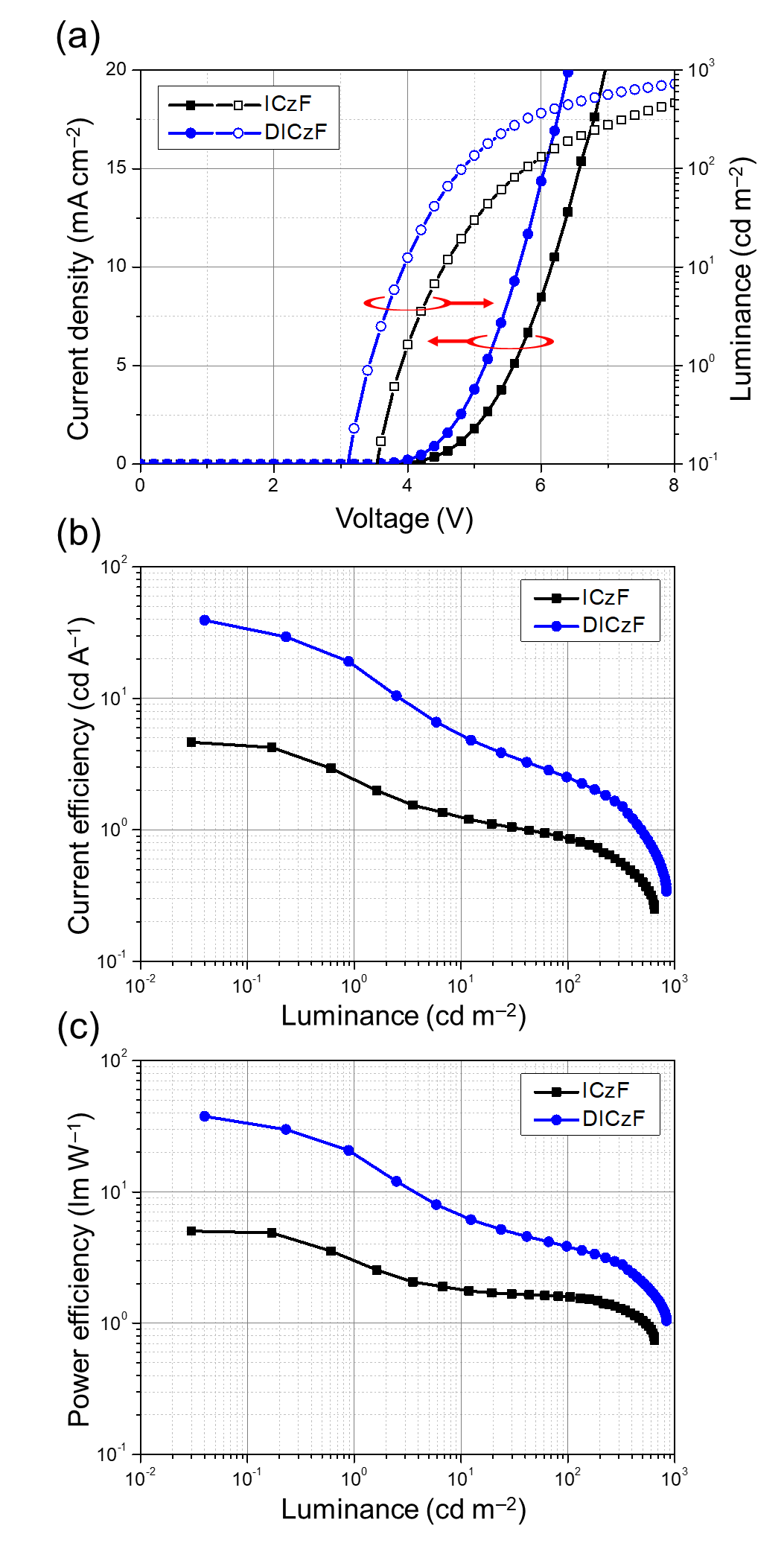


Figure S23. Device performance data of ICzF (black)- and DICzF (blue)-embedded blue MR–TADF devices. (a) Current density–voltage–luminance curves. (b) Current efficiency–luminance curves. (c) Power efficiency–luminance curves.


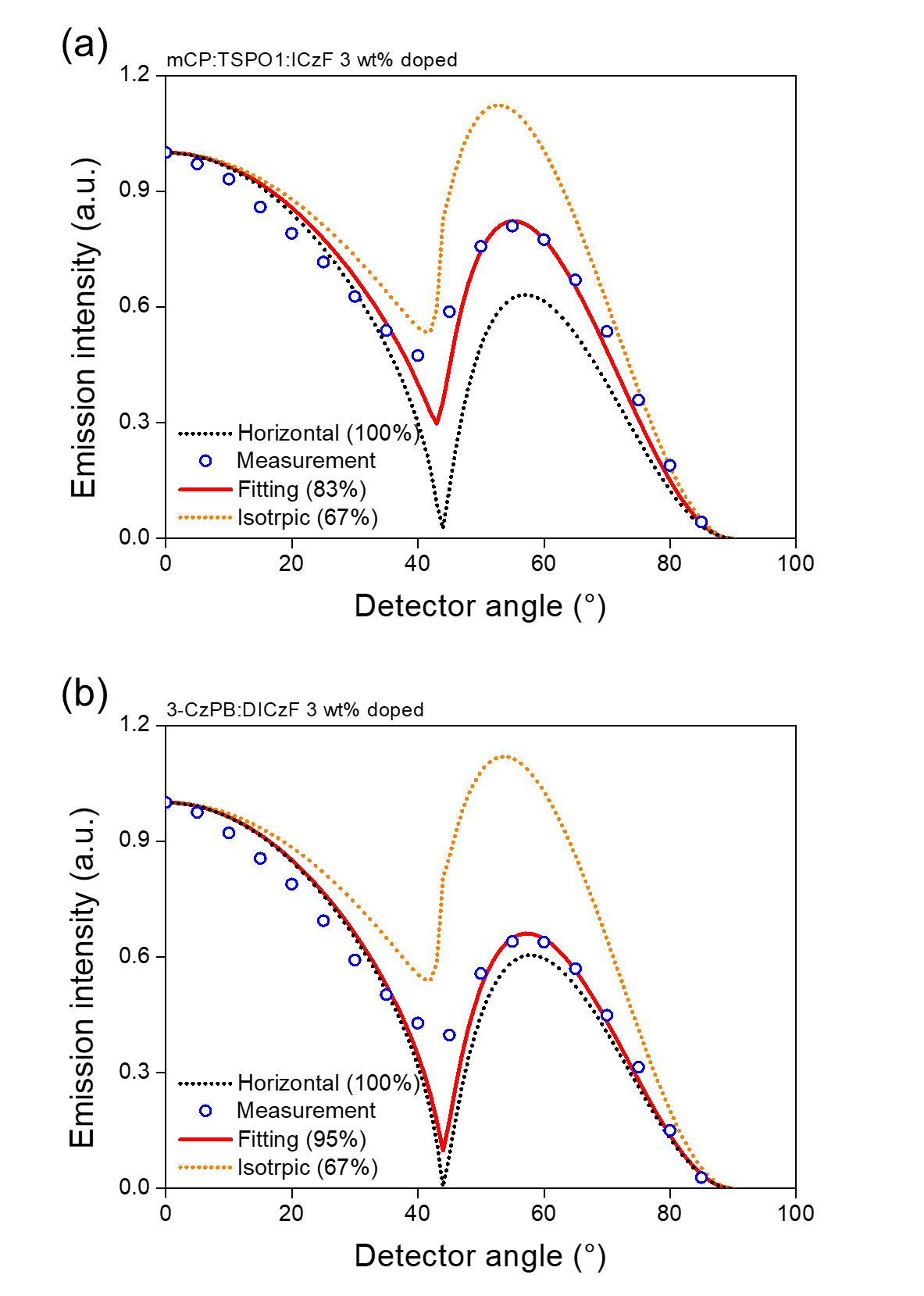


Figure S24. Angle-dependent PL measurement results of (a) mCP:TSPO1:ICzF film and (b) 3-CzPB:DICzF film.

**6. Supplementary tables**


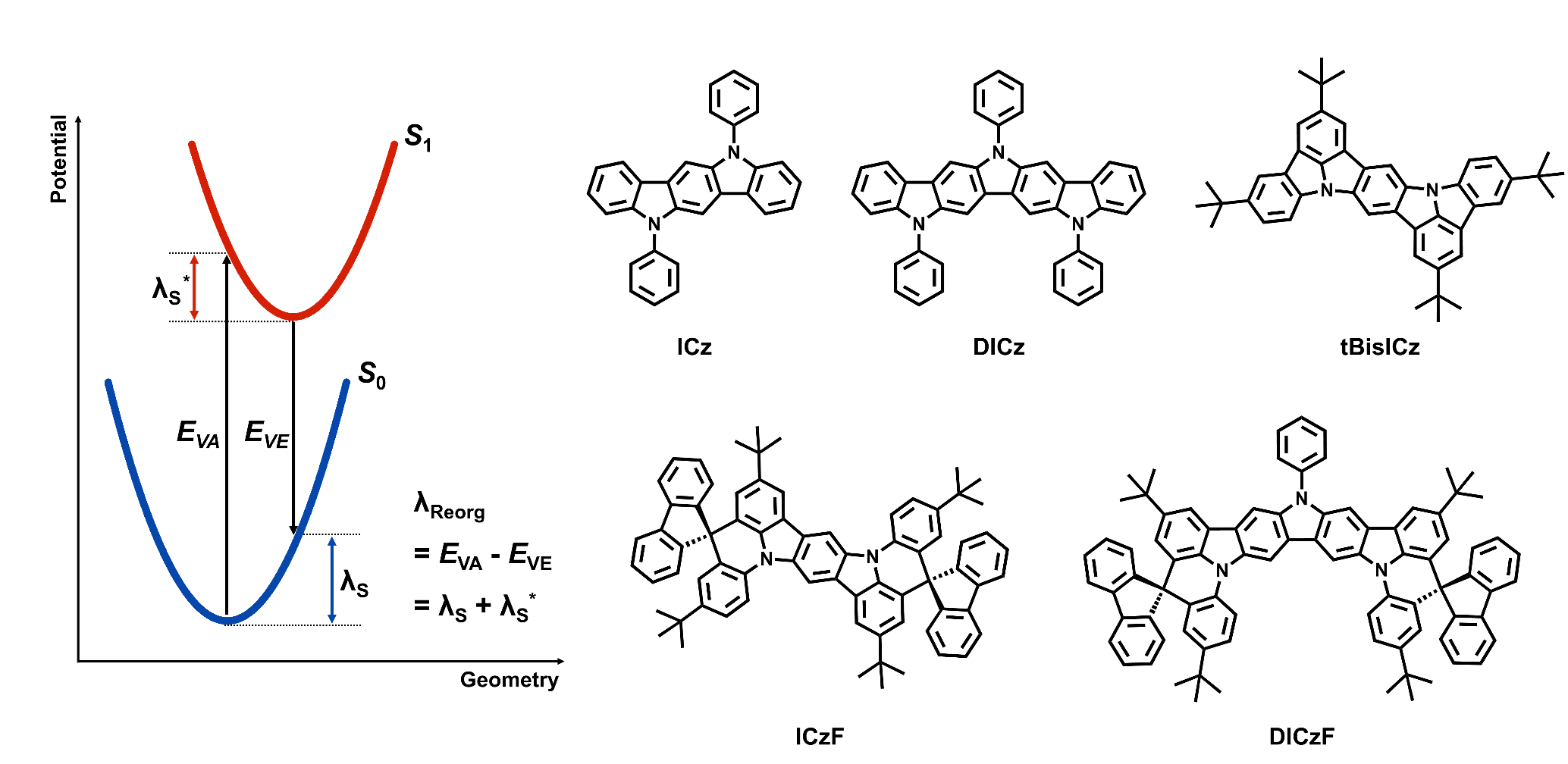


Table S1. The calculated material properties of tBisICz, ICzF, and DICzF.

|  | **B3LYP/6-31g(*d*) level** | | |
| --- | --- | --- | --- |
|  | **tBisICz** | **ICzF** | **DICzF** |
| **HOMO (eV)***^a^* | -5.0214 | -4.7298 | -4.6077 |
| **LUMO (eV)***^a^* | -1.4603 | -1.1595 | -1.3121 |
| ***E*_g_ (eV)***^a^* | 3.5611 | 3.5703 | 3.2955 |
| **S_1_ (eV)***^a^* | 3.0356 | 3.0543 | 2.8283 |
| **T_1_ (eV)***^a^* | 2.6324 | 2.6064 | 2.3808 |
| **Δ*E*_ST_ (eV)***^a^* | 0.4032 | 0.4479 | 0.4475 |
| **λ_S_ (eV)***^b^* | 0.1224 | 0.0988 | 0.0735 |
| **λ_S_^*^ (eV)***^c^* | 0.1024 | 0.0982 | 0.0741 |
| **λ_Reorg_ (eV)***^d^* | 0.2248 | 0.1970 | 0.1476 |

*^a^* Calculated at optimized S_0_ geometry.

*^b^* Ground state reorganization energy between S_0_ and S_1_ state at optimized S_0_ and S_1_ geometry.

*^c^* Excited state reorganization energy between S_0_ and S_1_ state at optimized S_0_ and S_1_ geometry.

*^d^* Total reorganization energy between S_0_ and S_1_ state.

Table S2. The Cartesian coordinates of optimized S_0_ geometry of ICz.

|  | **X (Å)** | **Y (Å)** | **Z (Å)** |
| --- | --- | --- | --- |
| ***Optimized S_0_ geometry*** | | | |
| C | -3.79149 | 0.731275 | -0.01904 |
| N | -2.75961 | -0.24389 | -0.03429 |
| C | -2.94333 | -1.62944 | -0.06946 |
| C | -4.12901 | -2.3676 | -0.12401 |
| C | -4.02852 | -3.75678 | -0.17301 |
| C | -2.78024 | -4.40233 | -0.17329 |
| C | -1.60233 | -3.66168 | -0.13215 |
| C | -1.6747 | -2.26472 | -0.08325 |
| C | -0.67541 | -1.21776 | -0.052 |
| C | -1.38018 | 0.021972 | -0.02556 |
| C | -0.72257 | 1.247169 | 0.032564 |
| C | 0.675408 | 1.217757 | 0.051522 |
| C | 1.38017 | -0.02197 | 0.025068 |
| N | 2.759598 | 0.24389 | 0.033712 |
| C | 3.791484 | -0.73129 | 0.019148 |
| C | 3.826301 | -1.73467 | 0.996578 |
| C | 4.83671 | -2.69563 | 0.973019 |
| C | 5.82847 | -2.65314 | -0.00901 |
| C | 5.798985 | -1.64726 | -0.97692 |
| C | 4.780436 | -0.69455 | -0.97265 |
| C | 2.943325 | 1.629438 | 0.068979 |
| C | 4.129004 | 2.367605 | 0.123537 |
| C | 4.028509 | 3.756779 | 0.17255 |
| C | 2.780228 | 4.402328 | 0.172817 |
| C | 1.602325 | 3.661679 | 0.13167 |
| C | 1.674697 | 2.264716 | 0.082771 |
| C | 0.722568 | -1.24717 | -0.03307 |
| C | -4.7801 | 0.694244 | 0.973098 |
| C | -5.79862 | 1.64697 | 0.978021 |
| C | -5.82843 | 2.653154 | 0.010438 |
| C | -4.83703 | 2.695922 | -0.97194 |
| C | -3.82665 | 1.734948 | -0.99616 |
| H | -5.09683 | -1.87735 | -0.13413 |
| H | -4.9378 | -4.35049 | -0.21477 |
| H | -2.73625 | -5.48708 | -0.21042 |
| H | -0.63675 | -4.16098 | -0.14322 |
| H | -1.26593 | 2.185749 | 0.061645 |
| H | 3.068342 | -1.74757 | 1.773559 |
| H | 4.855516 | -3.47106 | 1.73404 |
| H | 6.618554 | -3.39865 | -0.01995 |
| H | 6.563105 | -1.61027 | -1.74863 |
| H | 4.737826 | 0.073368 | -1.73875 |
| H | 5.096827 | 1.877358 | 0.133657 |
| H | 4.93779 | 4.350494 | 0.214318 |
| H | 2.736231 | 5.487084 | 0.20995 |
| H | 0.636742 | 4.160972 | 0.142727 |
| H | 1.26593 | -2.18575 | -0.06216 |
| H | -4.73725 | -0.07393 | 1.738927 |
| H | -6.56247 | 1.609758 | 1.749991 |
| H | -6.6185 | 3.39868 | 0.021885 |
| H | -4.85609 | 3.471582 | -1.73272 |
| H | -3.06898 | 1.748054 | -1.77342 |

Table S3. The Cartesian coordinates of optimized S_0_ geometry of DICz.

|  | **X (Å)** | **Y (Å)** | **Z (Å)** |
| --- | --- | --- | --- |
| ***Optimized S_0_ geometry*** | | | |
| C | 4.150705 | -2.57383 | 0.008608 |
| N | 4.148612 | -1.1543 | 0.005891 |
| C | 5.280898 | -0.33437 | -0.00362 |
| C | 6.633474 | -0.68641 | 0.013226 |
| C | 7.573351 | 0.342634 | 0.021071 |
| C | 7.182882 | 1.69265 | 0.017584 |
| C | 5.834318 | 2.037602 | 0.014134 |
| C | 4.869315 | 1.023636 | 0.007028 |
| C | 3.422007 | 1.028595 | 0.020367 |
| C | 2.481253 | 2.063958 | 0.017171 |
| C | 1.1381 | 1.700182 | 0.003539 |
| C | 0.723293 | 0.335016 | 0.007093 |
| C | 1.663676 | -0.70019 | 0.010466 |
| C | 3.006695 | -0.33544 | 0.02306 |
| C | -0.7237 | 0.334068 | 0.020195 |
| C | -1.14003 | 1.698688 | 0.027852 |
| C | -2.48319 | 2.061392 | -0.00183 |
| C | -3.42298 | 1.025215 | -0.00929 |
| C | -3.00652 | -0.3383 | 0.011365 |
| N | -4.14717 | -1.15863 | -0.01856 |
| C | -4.1468 | -2.57795 | -0.00696 |
| C | -3.48259 | -3.27369 | 1.012083 |
| C | -3.47761 | -4.66821 | 1.015186 |
| C | -4.14829 | -5.37889 | 0.017721 |
| C | -4.81772 | -4.6853 | -0.99235 |
| C | -4.8106 | -3.29085 | -1.01419 |
| C | -5.27982 | -0.34001 | -0.05856 |
| C | -6.63226 | -0.69305 | -0.06427 |
| C | -7.5729 | 0.335121 | -0.08512 |
| C | -7.18349 | 1.6854 | -0.09483 |
| C | -5.83538 | 2.031551 | -0.07498 |
| C | -4.86968 | 1.018481 | -0.05277 |
| C | -1.66313 | -0.70195 | 0.011298 |
| N | -0.0015 | 2.518633 | 0.019329 |
| C | -0.00361 | 3.937077 | 0.018997 |
| C | 0.684918 | 4.643634 | -0.97658 |
| C | 0.688434 | 6.038186 | -0.96671 |
| C | -0.00882 | 6.739233 | 0.019204 |
| C | -0.70383 | 6.035448 | 1.004811 |
| C | -0.69526 | 4.640968 | 1.014307 |
| C | 3.476672 | -3.27746 | 1.015637 |
| C | 3.474593 | -4.67202 | 1.009593 |
| C | 4.158021 | -5.37469 | 0.015139 |
| C | 4.837628 | -4.67322 | -0.98264 |
| C | 4.827739 | -3.27866 | -0.9954 |
| H | 6.944104 | -1.72589 | 0.025912 |
| H | 8.630649 | 0.091582 | 0.033031 |
| H | 7.940455 | 2.471167 | 0.022094 |
| H | 5.532564 | 3.081932 | 0.02222 |
| H | 2.790275 | 3.103823 | 0.024361 |
| H | 1.355005 | -1.74032 | 0.00311 |
| H | -2.79219 | 3.100965 | -0.03032 |
| H | -2.985 | -2.7166 | 1.799753 |
| H | -2.95947 | -5.19968 | 1.808881 |
| H | -4.14903 | -6.4652 | 0.027373 |
| H | -5.33661 | -5.23 | -1.77648 |
| H | -5.30653 | -2.74753 | -1.81243 |
| H | -6.94245 | -1.73258 | -0.04729 |
| H | -8.63003 | 0.083152 | -0.09146 |
| H | -7.94158 | 2.463194 | -0.11391 |
| H | -5.53456 | 3.076173 | -0.07258 |
| H | -1.35385 | -1.74179 | -0.00791 |
| H | 1.202443 | 4.095006 | -1.75728 |
| H | 1.226316 | 6.577193 | -1.74198 |
| H | -0.01074 | 7.825582 | 0.019348 |
| H | -1.24402 | 6.572342 | 1.779948 |
| H | -1.2118 | 4.090109 | 1.794118 |
| H | 2.96951 | -2.72651 | 1.80152 |
| H | 2.948701 | -5.2098 | 1.793882 |
| H | 4.160925 | -6.46104 | 0.017697 |
| H | 5.366783 | -5.21178 | -1.76414 |
| H | 5.33198 | -2.72889 | -1.78399 |

Table S4. The Cartesian coordinates of optimized S_0_ and S_1_ geometry of tBisICz.

|  | **X (Å)** | **Y (Å)** | **Z (Å)** |  | **X (Å)** | **Y (Å)** | **Z (Å)** |
| --- | --- | --- | --- | --- | --- | --- | --- |
| ***Optimized S_0_ geometry*** | | | | ***Optimized S_1_ geometry*** | | | |
| C | -5.288393 | 5.265928 | 1.263107 | C | -5.318215 | 5.261878 | 1.262893 |
| C | -4.431902 | 5.008001 | 0.000001 | C | -4.459382 | 5.011102 | 0.000004 |
| C | -5.289677 | 5.265782 | -1.262262 | C | -5.318665 | 5.261882 | -1.262578 |
| C | -3.278150 | 6.029729 | -0.000637 | C | -3.315748 | 6.044314 | -0.000199 |
| C | -3.933933 | 3.543798 | -0.000195 | C | -3.952165 | 3.548983 | -0.000092 |
| C | -4.914244 | 2.512108 | -0.000114 | C | -4.928615 | 2.519180 | -0.000053 |
| C | -4.522932 | 1.169097 | -0.000353 | C | -4.510278 | 1.165864 | -0.000150 |
| C | -3.151249 | 0.984355 | -0.000729 | C | -3.146004 | 1.006708 | -0.000296 |
| N | -2.750563 | -0.320154 | -0.000789 | N | -2.721296 | -0.317432 | -0.000304 |
| C | -3.919515 | -1.099605 | -0.000367 | C | -3.875544 | -1.104846 | -0.000150 |
| C | -5.047508 | -0.205537 | -0.000148 | C | -5.014181 | -0.211015 | -0.000063 |
| C | -6.332392 | -0.740984 | 0.000226 | C | -6.288278 | -0.769597 | 0.000090 |
| C | -6.540353 | -2.131474 | 0.000409 | C | -6.473658 | -2.167562 | 0.000157 |
| C | -7.980042 | -2.682539 | 0.000841 | C | -7.905096 | -2.734672 | 0.000342 |
| C | -8.724069 | -2.184785 | 1.263017 | C | -8.653386 | -2.242636 | 1.263162 |
| C | -8.724788 | -2.184881 | -1.260950 | C | -8.653627 | -2.242879 | -1.262430 |
| C | -8.020838 | -4.222635 | 0.000913 | C | -7.931386 | -4.275085 | 0.000491 |
| C | -5.410391 | -2.968821 | 0.000184 | C | -5.335932 | -2.999744 | 0.000065 |
| C | -4.101569 | -2.474858 | -0.000214 | C | -4.036530 | -2.484641 | -0.000094 |
| C | -1.343847 | -0.313001 | -0.000739 | C | -1.337641 | -0.309583 | -0.000300 |
| C | -0.447743 | -1.375102 | -0.000761 | C | -0.448014 | -1.386045 | -0.000308 |
| C | 0.915968 | -1.070212 | -0.000739 | C | 0.919740 | -1.102101 | -0.000308 |
| C | 1.343847 | 0.313001 | -0.000737 | C | 1.337641 | 0.309583 | -0.000300 |
| N | 2.750563 | 0.320154 | -0.000786 | N | 2.721296 | 0.317432 | -0.000303 |
| C | 3.919515 | 1.099605 | -0.000364 | C | 3.875544 | 1.104846 | -0.000149 |
| C | 4.101569 | 2.474858 | -0.000211 | C | 4.036530 | 2.484641 | -0.000093 |
| C | 5.410391 | 2.968821 | 0.000187 | C | 5.335932 | 2.999744 | 0.000066 |
| C | 6.540353 | 2.131474 | 0.000412 | C | 6.473658 | 2.167562 | 0.000158 |
| C | 7.980042 | 2.682539 | 0.000844 | C | 7.905096 | 2.734672 | 0.000343 |
| C | 8.724069 | 2.184785 | 1.263021 | C | 8.653386 | 2.242636 | 1.263163 |
| C | 8.724788 | 2.184880 | -1.260946 | C | 8.653627 | 2.242879 | -1.262429 |
| C | 8.020838 | 4.222635 | 0.000915 | C | 7.931386 | 4.275085 | 0.000491 |
| C | 6.332392 | 0.740984 | 0.000229 | C | 6.288278 | 0.769597 | 0.000091 |
| C | 5.047508 | 0.205537 | -0.000145 | C | 5.014181 | 0.211015 | -0.000062 |
| C | 4.522932 | -1.169097 | -0.000350 | C | 4.510278 | -1.165864 | -0.000149 |
| C | 3.151249 | -0.984355 | -0.000726 | C | 3.146004 | -1.006708 | -0.000295 |
| C | 2.122054 | -1.905899 | -0.000697 | C | 2.115653 | -1.925066 | -0.000293 |
| C | 2.549264 | -3.243741 | -0.000459 | C | 2.560072 | -3.271378 | -0.000198 |
| C | 3.933933 | -3.543798 | -0.000196 | C | 3.952165 | -3.548983 | -0.000092 |
| C | 4.431902 | -5.008001 | 0.000001 | C | 4.459382 | -5.011102 | 0.000004 |
| C | 5.289657 | -5.265791 | -1.262274 | C | 5.318656 | -5.261885 | -1.262583 |
| C | 5.288413 | -5.265919 | 1.263095 | C | 5.318224 | -5.261874 | 1.262888 |
| C | 3.278150 | -6.029729 | -0.000613 | C | 3.315748 | -6.044314 | -0.000187 |
| C | 4.914244 | -2.512108 | -0.000113 | C | 4.928615 | -2.519180 | -0.000052 |
| C | 0.447743 | 1.375102 | -0.000760 | C | 0.448014 | 1.386045 | -0.000308 |
| C | -0.915968 | 1.070212 | -0.000740 | C | -0.919740 | 1.102101 | -0.000308 |
| C | -2.122054 | 1.905899 | -0.000698 | C | -2.115653 | 1.925066 | -0.000293 |
| C | -2.549264 | 3.243741 | -0.000459 | C | -2.560072 | 3.271378 | -0.000199 |
| H | -5.650676 | 6.301454 | 1.275184 | H | -5.685932 | 6.295701 | 1.276807 |
| H | -4.701240 | 5.100329 | 2.173628 | H | -4.729783 | 5.097931 | 2.172945 |
| H | -6.162645 | 4.608224 | 1.307057 | H | -6.187418 | 4.597943 | 1.305246 |
| H | -5.652079 | 6.301270 | -1.274033 | H | -5.686421 | 6.295693 | -1.276338 |
| H | -6.163900 | 4.607975 | -1.305297 | H | -6.187861 | 4.597919 | -1.304642 |
| H | -4.703418 | 5.100191 | -2.173360 | H | -4.730548 | 5.097977 | -2.172841 |
| H | -3.688488 | 7.045865 | -0.000314 | H | -3.736146 | 7.056519 | -0.000023 |
| H | -2.644493 | 5.931341 | -0.889416 | H | -2.680960 | 5.950886 | -0.888534 |
| H | -2.643398 | 5.931213 | 0.887347 | H | -2.680526 | 5.950754 | 0.887814 |
| H | -5.965926 | 2.781875 | 0.000161 | H | -5.982233 | 2.775611 | 0.000052 |
| H | -7.182141 | -0.063890 | 0.000388 | H | -7.150439 | -0.109387 | 0.000164 |
| H | -8.771578 | -1.091491 | 1.304256 | H | -8.713920 | -1.150275 | 1.303646 |
| H | -8.223653 | -2.531194 | 2.174471 | H | -8.149620 | -2.583441 | 2.174768 |
| H | -9.753696 | -2.563350 | 1.274785 | H | -9.677977 | -2.634136 | 1.273639 |
| H | -8.772298 | -1.091589 | -1.302255 | H | -8.714190 | -1.150527 | -1.303105 |
| H | -9.754428 | -2.563427 | -1.272091 | H | -9.678213 | -2.634399 | -1.272645 |
| H | -8.224904 | -2.531378 | -2.172662 | H | -8.150024 | -2.583843 | -2.174066 |
| H | -7.536051 | -4.644235 | 0.888860 | H | -7.443526 | -4.692235 | 0.888726 |
| H | -7.536614 | -4.644304 | -0.887308 | H | -7.443622 | -4.692404 | -0.887717 |
| H | -9.062353 | -4.563397 | 0.001255 | H | -8.969850 | -4.624514 | 0.000582 |
| H | -5.539036 | -4.044984 | 0.000316 | H | -5.453497 | -4.076318 | 0.000113 |
| H | -3.257764 | -3.157773 | -0.000387 | H | -3.181290 | -3.153062 | -0.000167 |
| H | -0.789905 | -2.405039 | -0.000781 | H | -0.807624 | -2.409822 | -0.000306 |
| H | 3.257764 | 3.157773 | -0.000384 | H | 3.181290 | 3.153062 | -0.000166 |
| H | 5.539036 | 4.044984 | 0.000319 | H | 5.453497 | 4.076318 | 0.000114 |
| H | 8.771578 | 1.091492 | 1.304260 | H | 8.713920 | 1.150275 | 1.303648 |
| H | 8.223653 | 2.531196 | 2.174474 | H | 8.149620 | 2.583442 | 2.174769 |
| H | 9.753696 | 2.563351 | 1.274789 | H | 9.677977 | 2.634137 | 1.273640 |
| H | 8.772298 | 1.091588 | -1.302251 | H | 8.714190 | 1.150527 | -1.303103 |
| H | 9.754429 | 2.563426 | -1.272088 | H | 9.678213 | 2.634399 | -1.272645 |
| H | 8.224905 | 2.531376 | -2.172658 | H | 8.150024 | 2.583842 | -2.174066 |
| H | 7.536052 | 4.644235 | 0.888862 | H | 7.443525 | 4.692235 | 0.888725 |
| H | 7.536613 | 4.644303 | -0.887306 | H | 7.443622 | 4.692403 | -0.887717 |
| H | 9.062353 | 4.563397 | 0.001256 | H | 8.969850 | 4.624514 | 0.000582 |
| H | 7.182141 | 0.063890 | 0.000391 | H | 7.150439 | 0.109387 | 0.000165 |
| H | 1.825640 | -4.049989 | -0.000428 | H | 1.846005 | -4.084484 | -0.000192 |
| H | 6.163881 | -4.607987 | -1.305326 | H | 6.187853 | -4.597923 | -1.304654 |
| H | 4.703385 | -5.100203 | -2.173364 | H | 4.730533 | -5.097981 | -2.172842 |
| H | 5.652057 | -6.301280 | -1.274045 | H | 5.686410 | -6.295697 | -1.276343 |
| H | 6.162664 | -4.608212 | 1.307028 | H | 6.187427 | -4.597939 | 1.305233 |
| H | 5.650699 | -6.301445 | 1.275172 | H | 5.685942 | -6.295697 | 1.276801 |
| H | 4.701273 | -5.100316 | 2.173624 | H | 4.729798 | -5.097927 | 2.172944 |
| H | 2.644473 | -5.931340 | -0.889378 | H | 2.680950 | -5.950883 | -0.888515 |
| H | 2.643417 | -5.931214 | 0.887385 | H | 2.680537 | -5.950756 | 0.887833 |
| H | 3.688488 | -7.045865 | -0.000300 | H | 3.736146 | -7.056519 | -0.000018 |
| H | 5.965926 | -2.781875 | 0.000163 | H | 5.982233 | -2.775611 | 0.000053 |
| H | 0.789905 | 2.405039 | -0.000779 | H | 0.807624 | 2.409822 | -0.000306 |
| H | -1.825640 | 4.049989 | -0.000426 | H | -1.846005 | 4.084484 | -0.000192 |

Table S5. The Cartesian coordinates of optimized S_0_, S_1_ and T_1_ geometry of ICzF.

|  | **X (Å)** | **Y (Å)** | **Z (Å)** |  | **X (Å)** | **Y (Å)** | **Z (Å)** |  | **X (Å)** | **Y (Å)** | **Z (Å)** |
| --- | --- | --- | --- | --- | --- | --- | --- | --- | --- | --- | --- |
| ***Optimized S_0_ geometry*** | | | | ***Optimized S_1_ geometry*** | | | | ***Optimized T_1_ geometry*** | | | |
| C | 4.767065 | -5.486264 | 1.495234 | C | 4.730830 | -5.456352 | 1.628180 | C | 4.726347 | -5.456512 | 1.595287 |
| C | 4.017836 | -5.201451 | 0.171575 | C | 4.009431 | -5.207797 | 0.281804 | C | 3.996392 | -5.199945 | 0.255446 |
| C | 4.918507 | -5.598816 | -1.022336 | C | 4.932813 | -5.642469 | -0.881546 | C | 4.910730 | -5.628824 | -0.916727 |
| C | 2.761034 | -6.091789 | 0.136110 | C | 2.752465 | -6.098309 | 0.246963 | C | 2.736076 | -6.085624 | 0.223917 |
| C | 3.676028 | -3.700796 | 0.081797 | C | 3.678507 | -3.707582 | 0.142449 | C | 3.663731 | -3.699064 | 0.126661 |
| C | 2.358608 | -3.235364 | -0.012681 | C | 2.352953 | -3.252324 | 0.005693 | C | 2.344319 | -3.235115 | 0.000072 |
| C | 2.109649 | -1.860660 | -0.097012 | C | 2.095449 | -1.874952 | -0.117957 | C | 2.092315 | -1.844676 | -0.114984 |
| C | 0.919499 | -1.036521 | -0.182708 | C | 0.924072 | -1.057048 | -0.226252 | C | 0.929368 | -1.047658 | -0.206383 |
| C | -0.436789 | -1.372673 | -0.178758 | C | -0.452852 | -1.377284 | -0.234084 | C | -0.454125 | -1.396738 | -0.214783 |
| C | -1.363407 | -0.331846 | -0.221649 | C | -1.357240 | -0.316973 | -0.280294 | C | -1.368165 | -0.369430 | -0.250945 |
| C | -0.931319 | 1.032474 | -0.180229 | C | -0.937993 | 1.057522 | -0.226663 | C | -0.942714 | 1.043693 | -0.203296 |
| C | -2.121643 | 1.857448 | -0.091465 | C | -2.109811 | 1.875924 | -0.115437 | C | -2.105124 | 1.840717 | -0.109294 |
| C | -3.209816 | 0.966328 | -0.084051 | C | -3.216694 | 0.995776 | -0.114286 | C | -3.211875 | 0.954288 | -0.112275 |
| N | -2.772330 | -0.350320 | -0.196790 | N | -2.765046 | -0.325272 | -0.274675 | N | -2.764642 | -0.372585 | -0.248018 |
| C | -3.716631 | -1.358927 | -0.468682 | C | -3.699149 | -1.332560 | -0.510898 | C | -3.716324 | -1.387948 | -0.441660 |
| C | -3.343339 | -2.625097 | -0.936144 | C | -3.322366 | -2.598169 | -0.996228 | C | -3.355176 | -2.678634 | -0.853888 |
| C | -4.296031 | -3.608079 | -1.191006 | C | -4.265637 | -3.593834 | -1.204275 | C | -4.312264 | -3.673459 | -1.024139 |
| C | -5.661180 | -3.367615 | -1.004904 | C | -5.628469 | -3.372721 | -0.952955 | C | -5.672084 | -3.425075 | -0.802110 |
| C | -6.750647 | -4.423961 | -1.259153 | C | -6.707487 | -4.449292 | -1.149823 | C | -6.765568 | -4.495978 | -0.958278 |
| C | -7.552513 | -4.662780 | 0.042396 | C | -7.447589 | -4.683638 | 0.189374 | C | -7.519849 | -4.661649 | 0.382549 |
| C | -7.709177 | -3.923261 | -2.365920 | C | -7.720808 | -3.974490 | -2.219400 | C | -7.764254 | -4.059929 | -2.056901 |
| C | -6.157809 | -5.772009 | -1.711133 | C | -6.112512 | -5.792927 | -1.612134 | C | -6.183994 | -5.866447 | -1.354115 |
| C | -6.016331 | -2.080008 | -0.580512 | C | -5.990717 | -2.086382 | -0.526582 | C | -6.016547 | -2.116776 | -0.439229 |
| C | -5.094130 | -1.064821 | -0.318226 | C | -5.080080 | -1.052019 | -0.315225 | C | -5.090028 | -1.086051 | -0.265623 |
| C | -5.620546 | 0.313449 | 0.148387 | C | -5.613512 | 0.326477 | 0.128657 | C | -5.620594 | 0.317017 | 0.106113 |
| C | -6.147461 | 0.236634 | 1.595460 | C | -6.191384 | 0.251388 | 1.558481 | C | -6.269217 | 0.319119 | 1.505011 |
| C | -5.455883 | -0.104035 | 2.751334 | C | -5.533012 | -0.066800 | 2.739417 | C | -5.680743 | 0.026535 | 2.729070 |
| C | -6.138976 | -0.104436 | 3.972535 | C | -6.257609 | -0.071380 | 3.936428 | C | -6.461455 | 0.095592 | 3.888007 |
| C | -7.495743 | 0.233048 | 4.029984 | C | -7.621373 | 0.242208 | 3.944337 | C | -7.811769 | 0.456404 | 3.816343 |
| C | -8.192555 | 0.576067 | 2.869249 | C | -8.283695 | 0.565754 | 2.758145 | C | -8.404387 | 0.753636 | 2.587210 |
| C | -7.512206 | 0.576241 | 1.649730 | C | -7.562326 | 0.568733 | 1.562207 | C | -7.626561 | 0.683441 | 1.429269 |
| C | -7.973890 | 0.886331 | 0.289240 | C | -7.981024 | 0.865480 | 0.185038 | C | -7.968230 | 0.934904 | 0.021908 |
| C | -6.893268 | 0.731601 | -0.599074 | C | -6.867492 | 0.728301 | -0.664349 | C | -6.821096 | 0.721321 | -0.765072 |
| C | -7.055358 | 0.966406 | -1.959509 | C | -6.983162 | 0.960871 | -2.029526 | C | -6.864513 | 0.893646 | -2.143429 |
| C | -8.312411 | 1.355670 | -2.434649 | C | -8.228714 | 1.328522 | -2.549936 | C | -8.070032 | 1.279164 | -2.739481 |
| C | -9.389202 | 1.509072 | -1.553615 | C | -9.339324 | 1.462301 | -1.708611 | C | -9.213175 | 1.490588 | -1.959883 |
| C | -9.228509 | 1.276447 | -0.186108 | C | -9.224317 | 1.233080 | -0.335912 | C | -9.171345 | 1.320872 | -0.574380 |
| C | -4.527156 | 1.376291 | 0.030868 | C | -4.524906 | 1.387011 | 0.034359 | C | -4.509931 | 1.355848 | 0.027342 |
| C | -4.745277 | 2.756066 | 0.116994 | C | -4.757778 | 2.778487 | 0.170381 | C | -4.734606 | 2.765102 | 0.154956 |
| C | -3.694066 | 3.695366 | 0.098552 | C | -3.703715 | 3.703699 | 0.154859 | C | -3.688298 | 3.693799 | 0.143445 |
| C | -3.951195 | 5.213474 | 0.193755 | C | -3.954459 | 5.218360 | 0.296718 | C | -3.937495 | 5.209753 | 0.276726 |
| C | -3.245500 | 5.780512 | 1.448630 | C | -3.217771 | 5.748539 | 1.550319 | C | -3.209028 | 5.744580 | 1.532731 |
| C | -3.389544 | 5.911883 | -1.067684 | C | -3.418770 | 5.948841 | -0.958212 | C | -3.392846 | 5.933622 | -0.977730 |
| C | -5.450340 | 5.553829 | 0.297117 | C | -5.448906 | 5.562808 | 0.443819 | C | -5.433222 | 5.552869 | 0.412082 |
| C | -2.373177 | 3.226597 | -0.000703 | C | -2.371927 | 3.247437 | 0.012873 | C | -2.360387 | 3.227416 | 0.012333 |
| C | 0.424274 | 1.368851 | -0.175937 | C | 0.438507 | 1.378235 | -0.233472 | C | 0.441806 | 1.393015 | -0.210178 |
| C | 1.351112 | 0.328043 | -0.221353 | C | 1.344223 | 0.318141 | -0.279022 | C | 1.354922 | 0.365007 | -0.249141 |
| N | 2.759763 | 0.346223 | -0.195725 | N | 2.750027 | 0.325666 | -0.272692 | N | 2.751789 | 0.369349 | -0.245172 |
| C | 3.704121 | 1.353749 | -0.473622 | C | 3.686525 | 1.331862 | -0.512132 | C | 3.703231 | 1.383548 | -0.445020 |
| C | 3.329997 | 2.617612 | -0.946075 | C | 3.311565 | 2.596897 | -0.998553 | C | 3.341917 | 2.672652 | -0.861805 |
| C | 4.282560 | 3.598951 | -1.208114 | C | 4.256688 | 3.590159 | -1.212519 | C | 4.299347 | 3.665929 | -1.039809 |
| C | 5.648036 | 3.358616 | -1.024635 | C | 5.619585 | 3.366407 | -0.965934 | C | 5.659665 | 3.417477 | -0.821380 |
| C | 6.737359 | 4.413071 | -1.287237 | C | 6.700842 | 4.439794 | -1.169405 | C | 6.753599 | 4.486672 | -0.986275 |
| C | 7.543061 | 4.657935 | 0.010823 | C | 7.446364 | 4.676266 | 0.166327 | C | 7.512406 | 4.658465 | 0.351212 |
| C | 7.692423 | 3.906228 | -2.394209 | C | 7.709268 | 3.959917 | -2.241250 | C | 7.748438 | 4.044534 | -2.085942 |
| C | 6.143999 | 5.759180 | -1.744265 | C | 6.107579 | 5.783651 | -1.633291 | C | 6.171895 | 5.855668 | -1.386995 |
| C | 6.003895 | 2.072975 | -0.594569 | C | 5.979707 | 2.080500 | -0.537251 | C | 6.004148 | 2.110623 | -0.453264 |
| C | 5.081789 | 1.059773 | -0.324829 | C | 5.066950 | 1.048781 | -0.319628 | C | 5.077309 | 1.081517 | -0.271592 |
| C | 5.606299 | -0.315669 | 0.151609 | C | 5.598758 | -0.328762 | 0.130800 | C | 5.607013 | -0.319412 | 0.110455 |
| C | 6.114953 | -0.231865 | 1.605044 | C | 6.165243 | -0.249708 | 1.564803 | C | 6.239488 | -0.314234 | 1.516700 |
| C | 5.409019 | 0.113454 | 2.750811 | C | 5.498166 | 0.073129 | 2.739576 | C | 5.637180 | -0.016069 | 2.732635 |
| C | 6.077295 | 0.120093 | 3.980179 | C | 6.213386 | 0.080990 | 3.942222 | C | 6.404946 | -0.078711 | 3.900576 |
| C | 7.433603 | -0.215516 | 4.055689 | C | 7.576756 | -0.233646 | 3.961912 | C | 7.756328 | -0.438341 | 3.845865 |
| C | 8.144838 | -0.562911 | 2.905058 | C | 8.248035 | -0.561484 | 2.781926 | C | 8.362979 | -0.740775 | 2.624864 |
| C | 7.479336 | -0.569460 | 1.677426 | C | 7.535950 | -0.567736 | 1.580464 | C | 7.598050 | -0.677098 | 1.458021 |
| C | 6.888354 | -0.735269 | -0.578210 | C | 6.858454 | -0.733135 | -0.650976 | C | 6.817458 | -0.725820 | -0.745093 |
| C | 7.957972 | -0.885150 | 0.324149 | C | 7.965210 | -0.868921 | 0.207486 | C | 7.955818 | -0.934749 | 0.055772 |
| C | 9.218582 | -1.276824 | -0.133802 | C | 9.212260 | -1.239429 | -0.302446 | C | 9.165964 | -1.322502 | -0.525015 |
| C | 9.396284 | -1.516037 | -1.498070 | C | 9.337918 | -1.473135 | -1.673448 | C | 9.223680 | -1.498734 | -1.909133 |
| C | 8.330372 | -1.367680 | -2.393084 | C | 8.234074 | -1.340866 | -2.523913 | C | 8.089277 | -1.291932 | -2.702621 |
| C | 7.067325 | -0.976771 | -1.935402 | C | 6.984880 | -0.970192 | -2.014472 | C | 6.876790 | -0.904551 | -2.122081 |
| C | 3.195220 | -0.970954 | -0.087003 | C | 3.200701 | -0.996392 | -0.115785 | C | 3.197771 | -0.958455 | -0.114021 |
| C | 4.516020 | -1.381038 | 0.026162 | C | 4.510486 | -1.390201 | 0.030932 | C | 4.497977 | -1.360889 | 0.024126 |
| C | 4.730018 | -2.757075 | 0.104398 | C | 4.737343 | -2.778218 | 0.160364 | C | 4.716871 | -2.764563 | 0.143237 |
| H | 5.020204 | -6.551228 | 1.569674 | H | 4.975943 | -6.520214 | 1.739827 | H | 4.972849 | -6.520670 | 1.698698 |
| H | 4.147164 | -5.222529 | 2.359744 | H | 4.094568 | -5.163366 | 2.471165 | H | 4.096173 | -5.169191 | 2.444763 |
| H | 5.699357 | -4.916929 | 1.568303 | H | 5.663363 | -4.888252 | 1.702543 | H | 5.660039 | -4.889485 | 1.667134 |
| H | 5.178747 | -6.663273 | -0.967680 | H | 5.184313 | -6.707037 | -0.793196 | H | 5.162316 | -6.693760 | -0.835931 |
| H | 5.852538 | -5.027767 | -1.037353 | H | 5.869403 | -5.076215 | -0.891974 | H | 5.848588 | -5.064250 | -0.930586 |
| H | 4.404942 | -5.422833 | -1.974572 | H | 4.439926 | -5.488635 | -1.848480 | H | 4.411822 | -5.469545 | -1.879640 |
| H | 3.053917 | -7.145952 | 0.199203 | H | 3.043128 | -7.150431 | 0.347233 | H | 3.023529 | -7.139145 | 0.316422 |
| H | 2.194952 | -5.961968 | -0.793475 | H | 2.205869 | -5.994700 | -0.697298 | H | 2.183610 | -5.975717 | -0.716311 |
| H | 2.090438 | -5.885267 | 0.978128 | H | 2.065195 | -5.865454 | 1.068209 | H | 2.054901 | -5.855718 | 1.051189 |
| H | 1.526104 | -3.929881 | -0.021709 | H | 1.526612 | -3.952766 | -0.001494 | H | 1.510388 | -3.926669 | -0.006723 |
| H | -0.731481 | -2.410246 | -0.091842 | H | -0.763420 | -2.408465 | -0.139433 | H | -0.736150 | -2.436854 | -0.136072 |
| H | -2.304675 | -2.839633 | -1.144518 | H | -2.290216 | -2.783914 | -1.256654 | H | -2.323761 | -2.899637 | -1.087771 |
| H | -3.947983 | -4.570376 | -1.549767 | H | -3.922698 | -4.550374 | -1.581495 | H | -3.974608 | -4.653043 | -1.343672 |
| H | -8.033114 | -3.745945 | 0.399202 | H | -7.927814 | -3.770895 | 0.556487 | H | -7.989718 | -3.726512 | 0.704344 |
| H | -6.899854 | -5.030133 | 0.842609 | H | -6.755661 | -5.032324 | 0.964391 | H | -6.838321 | -4.983447 | 1.178289 |
| H | -8.339273 | -5.408834 | -0.125029 | H | -8.227815 | -5.443967 | 0.062558 | H | -8.310069 | -5.416356 | 0.285125 |
| H | -8.191577 | -2.979324 | -2.091760 | H | -8.209350 | -3.037475 | -1.933407 | H | -8.242178 | -3.104192 | -1.818296 |
| H | -8.499532 | -4.661608 | -2.549871 | H | -8.503196 | -4.729924 | -2.361442 | H | -8.556322 | -4.810192 | -2.172173 |
| H | -7.169812 | -3.761201 | -3.306263 | H | -7.225655 | -3.812590 | -3.183627 | H | -7.258807 | -3.947941 | -3.022973 |
| H | -5.482327 | -6.193340 | -0.957693 | H | -5.396296 | -6.193785 | -0.885754 | H | -5.480241 | -6.243115 | -0.602927 |
| H | -5.605278 | -5.679869 | -2.653287 | H | -5.606802 | -5.705186 | -2.580553 | H | -5.666335 | -5.827400 | -2.319482 |
| H | -6.965078 | -6.495366 | -1.872417 | H | -6.914450 | -6.530655 | -1.726282 | H | -6.993868 | -6.599087 | -1.444593 |
| H | -7.066903 | -1.840839 | -0.449201 | H | -7.039456 | -1.864810 | -0.357756 | H | -7.063207 | -1.870886 | -0.288512 |
| H | -4.401887 | -0.366041 | 2.711362 | H | -4.472284 | -0.303852 | 2.737766 | H | -4.631626 | -0.251484 | 2.787787 |
| H | -5.610372 | -0.369207 | 4.884339 | H | -5.755781 | -0.318676 | 4.868002 | H | -6.014918 | -0.132318 | 4.852132 |
| H | -8.012071 | 0.228116 | 4.986263 | H | -8.169875 | 0.236070 | 4.882514 | H | -8.404704 | 0.506393 | 4.725755 |
| H | -9.246652 | 0.837565 | 2.918931 | H | -9.342404 | 0.812564 | 2.770366 | H | -9.453308 | 1.035062 | 2.537385 |
| H | -6.219324 | 0.847840 | -2.643683 | H | -6.119804 | 0.861047 | -2.682102 | H | -5.976826 | 0.729814 | -2.748805 |
| H | -8.453539 | 1.540864 | -3.496088 | H | -8.333853 | 1.514287 | -3.615428 | H | -8.119006 | 1.416241 | -3.816365 |
| H | -10.359675 | 1.812590 | -1.937340 | H | -10.300232 | 1.749539 | -2.127312 | H | -10.142387 | 1.790250 | -2.437302 |
| H | -10.067978 | 1.398446 | 0.493686 | H | -10.089635 | 1.341583 | 0.313059 | H | -10.062012 | 1.487767 | 0.026134 |
| H | -5.768257 | 3.100509 | 0.208354 | H | -5.779101 | 3.114760 | 0.291381 | H | -5.758348 | 3.100202 | 0.262891 |
| H | -2.163434 | 5.614352 | 1.418893 | H | -2.137795 | 5.578829 | 1.492341 | H | -2.128530 | 5.573779 | 1.482905 |
| H | -3.630181 | 5.308735 | 2.359969 | H | -3.584713 | 5.252924 | 2.456321 | H | -3.582560 | 5.254319 | 2.438958 |
| H | -3.414233 | 6.861761 | 1.527436 | H | -3.381752 | 6.827811 | 1.661994 | H | -3.371198 | 6.824534 | 1.638180 |
| H | -2.312544 | 5.747837 | -1.179728 | H | -2.345893 | 5.783513 | -1.100322 | H | -2.318828 | 5.767097 | -1.111775 |
| H | -3.557596 | 6.994728 | -1.013267 | H | -3.582424 | 7.030335 | -0.870093 | H | -3.554800 | 7.015643 | -0.895757 |
| H | -3.879778 | 5.536701 | -1.973310 | H | -3.932256 | 5.599533 | -1.861326 | H | -3.900363 | 5.581367 | -1.883045 |
| H | -5.907342 | 5.119332 | 1.193328 | H | -5.886484 | 5.103595 | 1.337232 | H | -5.877091 | 5.096788 | 1.304115 |
| H | -6.010468 | 5.202922 | -0.576933 | H | -6.029286 | 5.237699 | -0.426823 | H | -6.007554 | 5.225206 | -0.461720 |
| H | -5.577194 | 6.640859 | 0.356322 | H | -5.569420 | 6.648376 | 0.536518 | H | -5.555293 | 6.638481 | 0.500360 |
| H | -1.543221 | 3.927602 | -0.006514 | H | -1.550185 | 3.956572 | 0.007859 | H | -1.530198 | 3.926555 | 0.009374 |
| H | 0.719244 | 2.406281 | -0.087044 | H | 0.749141 | 2.409377 | -0.138363 | H | 0.724732 | 2.432583 | -0.127438 |
| H | 2.290770 | 2.831356 | -1.152750 | H | 2.278916 | 2.785059 | -1.255675 | H | 2.309813 | 2.893303 | -1.093453 |
| H | 3.934108 | 4.559671 | -1.570670 | H | 3.914364 | 4.546678 | -1.590542 | H | 3.961406 | 4.644307 | -1.362822 |
| H | 8.024399 | 3.742699 | 0.370733 | H | 7.925505 | 3.763288 | 0.534292 | H | 7.982558 | 3.724539 | 0.676105 |
| H | 6.892871 | 5.029467 | 0.811112 | H | 6.757950 | 5.028653 | 0.942823 | H | 6.833678 | 4.984662 | 1.147559 |
| H | 8.329595 | 5.402853 | -0.162558 | H | 8.228106 | 5.434327 | 0.034879 | H | 8.302928 | 5.412057 | 0.247580 |
| H | 8.174954 | 2.963320 | -2.116736 | H | 8.196039 | 3.022157 | -1.954643 | H | 8.226113 | 3.089424 | -1.844316 |
| H | 8.482761 | 4.643099 | -2.584009 | H | 8.493398 | 4.712720 | -2.387984 | H | 8.540933 | 4.793408 | -2.207322 |
| H | 7.150312 | 3.739941 | -3.332232 | H | 7.210262 | 3.796942 | -3.203329 | H | 7.239793 | 3.928455 | -3.049859 |
| H | 5.470990 | 6.184718 | -0.990975 | H | 5.395009 | 6.188218 | -0.905361 | H | 5.470916 | 6.236610 | -0.635360 |
| H | 5.588679 | 5.662659 | -2.684336 | H | 5.597892 | 5.694321 | -2.599488 | H | 5.650968 | 5.812216 | -2.350417 |
| H | 6.951226 | 6.481217 | -1.911522 | H | 6.910853 | 6.519173 | -1.752576 | H | 6.982044 | 6.587215 | -1.483798 |
| H | 7.054660 | 1.834143 | -0.464537 | H | 7.028418 | 1.856750 | -0.370908 | H | 7.051066 | 1.864743 | -0.304358 |
| H | 4.355374 | 0.374294 | 2.697089 | H | 4.437747 | 0.311283 | 2.728799 | H | 4.587249 | 0.261280 | 2.778369 |
| H | 5.537306 | 0.388326 | 4.884253 | H | 5.704495 | 0.331787 | 4.869041 | H | 5.947351 | 0.153339 | 4.858511 |
| H | 7.938249 | -0.205669 | 5.018131 | H | 8.117933 | -0.224843 | 4.904331 | H | 8.339088 | -0.483247 | 4.762092 |
| H | 9.198588 | -0.822769 | 2.968659 | H | 9.306484 | -0.808899 | 2.803168 | H | 9.412770 | -1.021001 | 2.588097 |
| H | 10.049626 | -1.395059 | 0.556914 | H | 10.072332 | -1.346637 | 0.353710 | H | 10.049911 | -1.485680 | 0.086365 |
| H | 10.371570 | -1.820884 | -1.868285 | H | 10.301815 | -1.762671 | -2.083660 | H | 10.158481 | -1.799873 | -2.374569 |
| H | 8.484582 | -1.558191 | -3.451735 | H | 8.347361 | -1.530266 | -3.587938 | H | 8.150497 | -1.434178 | -3.778199 |
| H | 6.239767 | -0.862330 | -2.630462 | H | 6.126761 | -0.871492 | -2.674061 | H | 5.995925 | -0.744355 | -2.738264 |
| H | 5.752691 | -3.109666 | 0.194319 | H | 5.757856 | -3.124474 | 0.280123 | H | 5.739519 | -3.110023 | 0.250488 |

Table S6. The Cartesian coordinates of optimized S_0_, S_1_ and T_1_ geometry of DICzF.

|  | **X (Å)** | **Y (Å)** | **Z (Å)** |  | **X (Å)** | **Y (Å)** | **Z (Å)** |  | **X (Å)** | **Y (Å)** | **Z (Å)** |
| --- | --- | --- | --- | --- | --- | --- | --- | --- | --- | --- | --- |
| ***Optimized S_0_ geometry*** | | | | ***Optimized S_1_ geometry*** | | | | ***Optimized T_1_ geometry*** | | | |
| C | -6.583688 | -6.004672 | -0.560725 | C | -6.530457 | -6.005579 | -0.458041 | C | -6.531161 | -6.022614 | -0.406687 |
| C | -5.130809 | -5.597384 | -0.870571 | C | -5.093432 | -5.584173 | -0.818483 | C | -5.083591 | -5.614313 | -0.739025 |
| C | -4.198146 | -6.367442 | 0.094455 | C | -4.120188 | -6.322451 | 0.131868 | C | -4.138077 | -6.347190 | 0.242474 |
| C | -4.813924 | -6.013351 | -2.326909 | C | -4.813230 | -6.020746 | -2.276752 | C | -4.773185 | -6.071530 | -2.184441 |
| C | -4.899249 | -4.085254 | -0.706043 | C | -4.877101 | -4.067793 | -0.687364 | C | -4.861839 | -4.096675 | -0.621878 |
| C | -3.633139 | -3.530238 | -0.945387 | C | -3.623056 | -3.502208 | -0.978305 | C | -3.600727 | -3.540342 | -0.889764 |
| C | -3.389750 | -2.173173 | -0.786313 | C | -3.391609 | -2.143239 | -0.848412 | C | -3.365028 | -2.179090 | -0.770795 |
| C | -4.405994 | -1.300844 | -0.364526 | C | -4.407839 | -1.276210 | -0.404047 | C | -4.384514 | -1.301239 | -0.364137 |
| N | -4.182255 | 0.077046 | -0.182952 | N | -4.191717 | 0.097662 | -0.244264 | N | -4.165003 | 0.080322 | -0.220419 |
| C | -5.280209 | 0.929381 | -0.110737 | C | -5.301448 | 0.945832 | -0.133042 | C | -5.278565 | 0.930535 | -0.123698 |
| C | -4.877552 | 2.274063 | -0.200006 | C | -4.885798 | 2.292838 | -0.213998 | C | -4.871128 | 2.279803 | -0.198459 |
| C | -3.430347 | 2.252720 | -0.296556 | C | -3.451970 | 2.267607 | -0.329478 | C | -3.442894 | 2.267526 | -0.301533 |
| C | -2.494972 | 3.286459 | -0.362012 | C | -2.495227 | 3.300001 | -0.397349 | C | -2.501400 | 3.313536 | -0.367817 |
| C | -1.149891 | 2.928291 | -0.385709 | C | -1.158864 | 2.920003 | -0.424777 | C | -1.166435 | 2.957025 | -0.388097 |
| N | -0.014313 | 3.748929 | -0.424221 | N | -0.018144 | 3.727445 | -0.458676 | N | -0.037836 | 3.760234 | -0.425071 |
| C | -0.019918 | 5.165695 | -0.508456 | C | -0.023033 | 5.144882 | -0.530032 | C | -0.035892 | 5.179438 | -0.499833 |
| C | -0.707879 | 5.805293 | -1.548362 | C | -0.738785 | 5.791151 | -1.547188 | C | -0.717660 | 5.827271 | -1.537879 |
| C | -0.720784 | 7.197790 | -1.622575 | C | -0.746377 | 7.183489 | -1.611127 | C | -0.720257 | 7.220104 | -1.603501 |
| C | -0.034113 | 7.961594 | -0.676562 | C | -0.037251 | 7.937244 | -0.673315 | C | -0.033219 | 7.972735 | -0.648772 |
| C | 0.658731 | 7.323727 | 0.354276 | C | 0.678575 | 7.290535 | 0.336507 | C | 0.652248 | 7.324656 | 0.380675 |
| C | 0.659527 | 5.932201 | 0.447849 | C | 0.685039 | 5.898894 | 0.415700 | C | 0.647262 | 5.932588 | 0.463633 |
| C | -0.734838 | 1.569772 | -0.297146 | C | -0.727661 | 1.555480 | -0.338823 | C | -0.719524 | 1.569566 | -0.303140 |
| C | 0.712024 | 1.573008 | -0.279982 | C | 0.701309 | 1.558531 | -0.318707 | C | 0.688905 | 1.582908 | -0.283553 |
| C | 1.123910 | 2.933729 | -0.356337 | C | 1.128504 | 2.923064 | -0.392587 | C | 1.114679 | 2.943680 | -0.360850 |
| C | 2.468828 | 3.292481 | -0.369680 | C | 2.463098 | 3.303668 | -0.412528 | C | 2.441647 | 3.314676 | -0.391595 |
| C | 3.406578 | 2.260075 | -0.308182 | C | 3.423273 | 2.272001 | -0.342575 | C | 3.414024 | 2.256259 | -0.328942 |
| C | 4.854318 | 2.283913 | -0.235556 | C | 4.857240 | 2.300988 | -0.251540 | C | 4.833556 | 2.291052 | -0.243841 |
| C | 5.259010 | 0.944104 | -0.132292 | C | 5.276184 | 0.958580 | -0.153490 | C | 5.260002 | 0.941525 | -0.144907 |
| N | 4.162980 | 0.087261 | -0.176659 | N | 4.169004 | 0.105042 | -0.232489 | N | 4.167377 | 0.090693 | -0.204360 |
| C | 4.387993 | -1.290891 | -0.360773 | C | 4.389328 | -1.269512 | -0.394418 | C | 4.390078 | -1.289850 | -0.373727 |
| C | 3.376139 | -2.165843 | -0.775243 | C | 3.378556 | -2.141813 | -0.827731 | C | 3.378136 | -2.163693 | -0.790049 |
| C | 3.622420 | -3.526363 | -0.941287 | C | 3.615655 | -3.504371 | -0.965353 | C | 3.621122 | -3.526257 | -0.939621 |
| C | 4.890109 | -4.071941 | -0.716256 | C | 4.873193 | -4.057925 | -0.692754 | C | 4.885694 | -4.072934 | -0.696850 |
| C | 5.899960 | -3.172409 | -0.348360 | C | 5.884209 | -3.162347 | -0.316716 | C | 5.896556 | -3.173151 | -0.331934 |
| C | 5.696555 | -1.802007 | -0.175319 | C | 5.692880 | -1.787495 | -0.174732 | C | 5.696768 | -1.800256 | -0.175569 |
| C | 5.209708 | -5.568916 | -0.871966 | C | 5.180744 | -5.559839 | -0.813076 | C | 5.201382 | -5.572522 | -0.833599 |
| C | 6.282171 | -5.757588 | -1.971330 | C | 6.278382 | -5.778699 | -1.881967 | C | 6.281002 | -5.776834 | -1.923161 |
| C | 5.747590 | -6.124846 | 0.468060 | C | 5.680429 | -6.093929 | 0.550775 | C | 5.728079 | -6.114390 | 0.516661 |
| C | 3.967359 | -6.389548 | -1.267015 | C | 3.940470 | -6.376414 | -1.222917 | C | 3.959261 | -6.393863 | -1.227918 |
| C | 6.584888 | 0.558582 | 0.005565 | C | 6.589347 | 0.568376 | 0.010603 | C | 6.582251 | 0.562542 | 0.001719 |
| C | 6.897561 | -0.919837 | 0.239992 | C | 6.897591 | -0.909658 | 0.232254 | C | 6.900162 | -0.915545 | 0.227788 |
| C | 8.197376 | -1.337428 | -0.458813 | C | 8.184986 | -1.333010 | -0.489000 | C | 8.189889 | -1.329784 | -0.492343 |
| C | 8.482920 | -1.336162 | -1.819268 | C | 8.452276 | -1.317282 | -1.852855 | C | 8.455851 | -1.319641 | -1.856640 |
| C | 9.750427 | -1.747307 | -2.245850 | C | 9.708536 | -1.739157 | -2.301517 | C | 9.716589 | -1.729363 | -2.304022 |
| C | 10.716870 | -2.150750 | -1.317161 | C | 10.681628 | -2.167649 | -1.391151 | C | 10.695714 | -2.140250 | -1.392006 |
| C | 10.433730 | -2.150205 | 0.050263 | C | 10.416838 | -2.180837 | -0.020115 | C | 10.432319 | -2.148297 | -0.020653 |
| C | 9.167769 | -1.741145 | 0.477215 | C | 9.162273 | -1.760457 | 0.429007 | C | 9.173142 | -1.740508 | 0.427074 |
| C | 8.592125 | -1.637890 | 1.825577 | C | 8.607036 | -1.663934 | 1.786371 | C | 8.617273 | -1.644071 | 1.784253 |
| C | 9.139285 | -1.922804 | 3.078362 | C | 9.168480 | -1.966708 | 3.028736 | C | 9.182302 | -1.935772 | 3.027573 |
| C | 8.353193 | -1.731456 | 4.216746 | C | 8.401575 | -1.774824 | 4.180040 | C | 8.413168 | -1.748837 | 4.178210 |
| C | 7.039798 | -1.262129 | 4.104317 | C | 7.093418 | -1.286069 | 4.091101 | C | 7.099036 | -1.276748 | 4.087363 |
| C | 6.490411 | -0.975687 | 2.849679 | C | 6.530046 | -0.980970 | 2.847127 | C | 6.531940 | -0.983325 | 2.842297 |
| C | 7.270939 | -1.165400 | 1.716001 | C | 7.290880 | -1.173102 | 1.700820 | C | 7.295340 | -1.169067 | 1.696422 |
| C | 7.529879 | 1.584684 | 0.005943 | C | 7.546522 | 1.596125 | 0.047209 | C | 7.525115 | 1.594722 | 0.020673 |
| C | 7.180837 | 2.950356 | -0.114411 | C | 7.191210 | 2.956950 | -0.069692 | C | 7.161742 | 2.965580 | -0.093096 |
| C | 8.301176 | 4.009328 | -0.107217 | C | 8.307325 | 4.020441 | -0.026614 | C | 8.279333 | 4.026486 | -0.065217 |
| C | 9.264133 | 3.750786 | -1.290657 | C | 9.299213 | 3.774878 | -1.188867 | C | 9.255666 | 3.778235 | -1.240148 |
| C | 7.753441 | 5.442714 | -0.244268 | C | 7.762199 | 5.454975 | -0.163634 | C | 7.732652 | 5.460674 | -0.196733 |
| C | 9.089382 | 3.924540 | 1.221746 | C | 9.063691 | 3.925181 | 1.320115 | C | 9.052637 | 3.931437 | 1.272027 |
| C | 5.826912 | 3.290164 | -0.226837 | C | 5.837522 | 3.305101 | -0.213140 | C | 5.815011 | 3.305902 | -0.217051 |
| C | 3.000775 | 0.884671 | -0.254325 | C | 3.008103 | 0.897247 | -0.297448 | C | 3.003267 | 0.888454 | -0.279402 |
| C | 1.651754 | 0.537169 | -0.214996 | C | 1.660643 | 0.526202 | -0.254683 | C | 1.656695 | 0.525926 | -0.219821 |
| C | -1.672611 | 0.532311 | -0.227172 | C | -1.685049 | 0.523046 | -0.272442 | C | -1.672882 | 0.518266 | -0.244884 |
| C | -3.022422 | 0.877538 | -0.262117 | C | -3.033691 | 0.893103 | -0.309215 | C | -3.010959 | 0.869399 | -0.276942 |
| C | -5.850638 | 3.272598 | -0.158404 | C | -5.867017 | 3.286705 | -0.138652 | C | -5.853043 | 3.277415 | -0.128895 |
| C | -7.207023 | 2.929651 | -0.027255 | C | -7.223383 | 2.931403 | 0.026342 | C | -7.211178 | 2.924426 | 0.024495 |
| C | -8.268236 | 4.048455 | 0.014010 | C | -8.281131 | 4.050626 | 0.106970 | C | -8.271429 | 4.041757 | 0.099197 |
| C | -7.989379 | 4.982261 | 1.215879 | C | -7.967434 | 4.972348 | 1.309869 | C | -7.969434 | 4.961291 | 1.306498 |
| C | -8.204042 | 4.869286 | -1.296174 | C | -8.250404 | 4.883650 | -1.196962 | C | -8.232204 | 4.877639 | -1.202419 |
| C | -9.700467 | 3.499776 | 0.160724 | C | -9.710502 | 3.504718 | 0.287387 | C | -9.700198 | 3.490558 | 0.267022 |
| C | -7.552483 | 1.566726 | 0.073055 | C | -7.573003 | 1.574222 | 0.121741 | C | -7.551750 | 1.567508 | 0.114056 |
| C | -6.600596 | 0.541005 | 0.038545 | C | -6.607349 | 0.548789 | 0.045627 | C | -6.582873 | 0.536606 | 0.044692 |
| C | -6.914853 | -0.941849 | 0.241215 | C | -6.914118 | -0.933463 | 0.232299 | C | -6.903093 | -0.945051 | 0.218580 |
| C | -7.319158 | -1.216251 | 1.703402 | C | -7.336817 | -1.228549 | 1.686542 | C | -7.380151 | -1.234263 | 1.655823 |
| C | -6.562233 | -1.048143 | 2.856340 | C | -6.599310 | -1.058740 | 2.851444 | C | -6.680987 | -1.076015 | 2.845894 |
| C | -7.137133 | -1.358204 | 4.093777 | C | -7.186885 | -1.390320 | 4.077278 | C | -7.315431 | -1.398410 | 4.050560 |
| C | -8.452542 | -1.829400 | 4.170089 | C | -8.495673 | -1.882831 | 4.129550 | C | -8.632821 | -1.870496 | 4.057438 |
| C | -9.215065 | -1.999003 | 3.012376 | C | -9.239096 | -2.052056 | 2.959453 | C | -9.337821 | -2.028664 | 2.862288 |
| C | -8.642295 | -1.690452 | 1.776688 | C | -8.653565 | -1.722814 | 1.735032 | C | -8.705241 | -1.708496 | 1.659015 |
| C | -9.189854 | -1.767909 | 0.414876 | C | -9.181179 | -1.792099 | 0.365025 | C | -9.185279 | -1.772128 | 0.271175 |
| C | -8.200170 | -1.346516 | -0.492708 | C | -8.186502 | -1.344392 | -0.524104 | C | -8.152598 | -1.341481 | -0.582506 |
| C | -8.456963 | -1.319240 | -1.858487 | C | -8.425605 | -1.300758 | -1.892441 | C | -8.341576 | -1.299728 | -1.958763 |
| C | -9.715139 | -1.721662 | -2.319349 | C | -9.671735 | -1.714846 | -2.375115 | C | -9.575445 | -1.697112 | -2.484942 |
| C | -10.700861 | -2.142517 | -1.419081 | C | -10.662329 | -2.163339 | -1.493764 | C | -10.603991 | -2.127419 | -1.638670 |
| C | -10.446495 | -2.168360 | -0.046259 | C | -10.425525 | -2.204701 | -0.118173 | C | -10.417468 | -2.167817 | -0.255312 |
| C | -5.707131 | -1.816820 | -0.170244 | C | -5.702768 | -1.801714 | -0.172169 | C | -5.682516 | -1.818560 | -0.149806 |
| C | -5.908697 | -3.193625 | -0.335130 | C | -5.889060 | -3.182964 | -0.305331 | C | -5.874680 | -3.201587 | -0.271374 |
| H | -6.697466 | -7.087562 | -0.685614 | H | -6.631076 | -7.092077 | -0.559097 | H | -6.637181 | -7.109632 | -0.497444 |
| H | -7.297271 | -5.517619 | -1.234829 | H | -7.270679 | -5.542589 | -1.120155 | H | -7.252979 | -5.562173 | -1.090630 |
| H | -6.864973 | -5.759146 | 0.469581 | H | -6.785093 | -5.744595 | 0.575327 | H | -6.807411 | -5.747664 | 0.617512 |
| H | -4.341605 | -7.449456 | -0.016313 | H | -4.252294 | -7.407777 | 0.043824 | H | -4.274047 | -7.433016 | 0.165318 |
| H | -4.407665 | -6.101267 | 1.136728 | H | -4.302322 | -6.041071 | 1.175207 | H | -4.342673 | -6.051320 | 1.277676 |
| H | -3.142279 | -6.150211 | -0.099547 | H | -3.073837 | -6.094817 | -0.097996 | H | -3.085303 | -6.128003 | 0.034519 |
| H | -4.965062 | -7.091996 | -2.458258 | H | -4.954319 | -7.103250 | -2.383310 | H | -4.917170 | -7.154688 | -2.281590 |
| H | -3.777447 | -5.787647 | -2.599736 | H | -3.788205 | -5.787791 | -2.584159 | H | -3.740279 | -5.846935 | -2.471246 |
| H | -5.467067 | -5.489743 | -3.034270 | H | -5.493937 | -5.518855 | -2.973657 | H | -5.435095 | -5.574614 | -2.902786 |
| H | -2.811815 | -4.161777 | -1.272651 | H | -2.808084 | -4.130169 | -1.326472 | H | -2.778887 | -4.175824 | -1.207595 |
| H | -2.412174 | -1.784154 | -1.032853 | H | -2.432085 | -1.739321 | -1.137640 | H | -2.394443 | -1.788213 | -1.039628 |
| H | -2.808727 | 4.325014 | -0.372967 | H | -2.795104 | 4.341741 | -0.406247 | H | -2.817789 | 4.350503 | -0.380371 |
| H | -1.217947 | 5.206507 | -2.296511 | H | -1.268521 | 5.197990 | -2.285331 | H | -1.230347 | 5.235349 | -2.289369 |
| H | -1.257719 | 7.685469 | -2.431682 | H | -1.299522 | 7.679218 | -2.404000 | H | -1.250778 | 7.716690 | -2.411273 |
| H | -0.039300 | 9.045930 | -0.741987 | H | -0.042393 | 9.022052 | -0.729154 | H | -0.031992 | 9.057488 | -0.706744 |
| H | 1.189616 | 7.910277 | 1.099191 | H | 1.226388 | 7.870341 | 1.074116 | H | 1.183915 | 7.903328 | 1.131009 |
| H | 1.172743 | 5.433275 | 1.263950 | H | 1.219572 | 5.390218 | 1.211093 | H | 1.158402 | 5.423441 | 1.274399 |
| H | 2.782924 | 4.329785 | -0.415369 | H | 2.763601 | 4.343139 | -0.478050 | H | 2.754063 | 4.350048 | -0.463975 |
| H | 2.394427 | -1.782400 | -1.013430 | H | 2.412350 | -1.745833 | -1.105403 | H | 2.401894 | -1.775070 | -1.043312 |
| H | 2.798976 | -4.154993 | -1.261776 | H | 2.798227 | -4.131130 | -1.303773 | H | 2.799456 | -4.155875 | -1.262478 |
| H | 6.906196 | -3.547473 | -0.190627 | H | 6.882588 | -3.544928 | -0.130533 | H | 6.900652 | -3.549765 | -0.164460 |
| H | 7.207529 | -5.222265 | -1.734636 | H | 7.203403 | -5.249137 | -1.631970 | H | 7.206640 | -5.242384 | -1.685784 |
| H | 5.920711 | -5.387394 | -2.937636 | H | 5.945047 | -5.422910 | -2.863600 | H | 5.927818 | -5.416094 | -2.896059 |
| H | 6.530282 | -6.820218 | -2.085250 | H | 6.516981 | -6.845839 | -1.969858 | H | 6.525820 | -6.841561 | -2.023472 |
| H | 6.657394 | -5.606811 | 0.788628 | H | 6.586823 | -5.578121 | 0.883934 | H | 6.637107 | -5.595433 | 0.837822 |
| H | 5.987284 | -7.191011 | 0.370691 | H | 5.912298 | -7.163699 | 0.478633 | H | 5.965190 | -7.182257 | 0.432959 |
| H | 5.003059 | -6.014737 | 1.264794 | H | 4.917503 | -5.962623 | 1.326567 | H | 4.978211 | -5.993173 | 1.306742 |
| H | 3.557261 | -6.070759 | -2.232243 | H | 3.558648 | -6.074787 | -2.205084 | H | 3.557523 | -6.085864 | -2.200130 |
| H | 3.173110 | -6.313737 | -0.515404 | H | 3.128044 | -6.277394 | -0.493777 | H | 3.159651 | -6.306287 | -0.483303 |
| H | 4.236807 | -7.447931 | -1.357423 | H | 4.201863 | -7.438805 | -1.284041 | H | 4.226013 | -7.454087 | -1.303215 |
| H | 7.733013 | -1.022225 | -2.540466 | H | 7.697880 | -0.981597 | -2.559448 | H | 7.696349 | -0.998961 | -2.564719 |
| H | 9.986390 | -1.752664 | -3.306572 | H | 9.931062 | -1.731989 | -3.365144 | H | 9.937584 | -1.727347 | -3.367989 |
| H | 11.697318 | -2.466941 | -1.663486 | H | 11.653314 | -2.491626 | -1.754596 | H | 11.670758 | -2.455107 | -1.754464 |
| H | 11.188634 | -2.463146 | 0.767133 | H | 11.177250 | -2.512118 | 0.682589 | H | 11.197178 | -2.466530 | 0.683246 |
| H | 10.159204 | -2.287817 | 3.170632 | H | 10.185248 | -2.344410 | 3.103104 | H | 10.203090 | -2.302202 | 3.103226 |
| H | 8.765292 | -1.949318 | 5.198475 | H | 8.825198 | -2.006075 | 5.153792 | H | 8.839310 | -1.971884 | 5.152771 |
| H | 6.440322 | -1.118537 | 4.999262 | H | 6.509627 | -1.140608 | 4.996060 | H | 6.513168 | -1.136100 | 4.991751 |
| H | 5.469951 | -0.611042 | 2.767332 | H | 5.515208 | -0.597365 | 2.782609 | H | 5.511467 | -0.615277 | 2.776393 |
| H | 8.575780 | 1.314183 | 0.111957 | H | 8.588514 | 1.323451 | 0.175917 | H | 8.571389 | 1.332180 | 0.136171 |
| H | 8.734530 | 3.821893 | -2.247696 | H | 8.791417 | 3.851007 | -2.157297 | H | 8.736110 | 3.856464 | -2.202114 |
| H | 9.720859 | 2.757221 | -1.235929 | H | 9.757837 | 2.782625 | -1.130818 | H | 9.713568 | 2.785194 | -1.188820 |
| H | 10.074038 | 4.490992 | -1.294017 | H | 10.105473 | 4.519023 | -1.167781 | H | 10.063861 | 4.520258 | -1.228436 |
| H | 7.205007 | 5.582079 | -1.182934 | H | 7.236337 | 5.602249 | -1.113812 | H | 7.194384 | 5.606761 | -1.140140 |
| H | 8.584374 | 6.157275 | -0.238362 | H | 8.592559 | 6.169718 | -0.130861 | H | 8.563245 | 6.175433 | -0.175847 |
| H | 7.086655 | 5.705892 | 0.584916 | H | 7.074991 | 5.709536 | 0.651309 | H | 7.056435 | 5.716265 | 0.627048 |
| H | 9.893352 | 4.671008 | 1.237889 | H | 9.864334 | 4.674437 | 1.362999 | H | 9.855184 | 4.679028 | 1.302685 |
| H | 8.432393 | 4.113259 | 2.078503 | H | 8.385063 | 4.103578 | 2.162124 | H | 8.385512 | 4.113116 | 2.122443 |
| H | 9.546741 | 2.940033 | 1.363898 | H | 9.519648 | 2.940482 | 1.463690 | H | 9.509444 | 2.946547 | 1.412121 |
| H | 5.521242 | 4.327150 | -0.308602 | H | 5.539394 | 4.343895 | -0.292488 | H | 5.508146 | 4.342492 | -0.292985 |
| H | 1.317192 | -0.485684 | -0.104368 | H | 1.342347 | -0.499149 | -0.128296 | H | 1.328590 | -0.493593 | -0.073888 |
| H | -1.336947 | -0.488980 | -0.105050 | H | -1.366234 | -0.500697 | -0.134161 | H | -1.336335 | -0.501448 | -0.125171 |
| H | -5.553673 | 4.315443 | -0.225886 | H | -5.580414 | 4.332085 | -0.201489 | H | -5.561948 | 4.321924 | -0.187206 |
| H | -6.999412 | 5.446085 | 1.151429 | H | -6.978640 | 5.434542 | 1.223782 | H | -6.980731 | 5.425700 | 1.229443 |
| H | -8.036969 | 4.428251 | 2.160288 | H | -7.991185 | 4.408746 | 2.249497 | H | -7.999887 | 4.395950 | 2.244889 |
| H | -8.732873 | 5.788184 | 1.255836 | H | -8.708656 | 5.778651 | 1.377328 | H | -8.712006 | 5.766574 | 1.369669 |
| H | -7.220466 | 5.328191 | -1.442397 | H | -7.270377 | 5.341709 | -1.365953 | H | -7.251631 | 5.338087 | -1.362739 |
| H | -8.948640 | 5.674990 | -1.280726 | H | -8.993398 | 5.689925 | -1.153375 | H | -8.976355 | 5.682938 | -1.162974 |
| H | -8.408209 | 4.234147 | -2.165774 | H | -8.479667 | 4.256496 | -2.066115 | H | -8.453648 | 4.252354 | -2.074930 |
| H | -9.828583 | 2.935324 | 1.091197 | H | -9.813731 | 2.931005 | 1.215192 | H | -9.808979 | 2.914010 | 1.192493 |
| H | -9.976044 | 2.847546 | -0.675639 | H | -10.009686 | 2.861321 | -0.547589 | H | -9.991109 | 2.848643 | -0.572073 |
| H | -10.412977 | 4.332400 | 0.179323 | H | -10.419980 | 4.338953 | 0.333776 | H | -10.412800 | 4.322220 | 0.310219 |
| H | -8.592274 | 1.285994 | 0.189650 | H | -8.607459 | 1.288508 | 0.263321 | H | -8.586672 | 1.276858 | 0.244790 |
| H | -5.540439 | -0.681905 | 2.801508 | H | -5.584178 | -0.672117 | 2.815074 | H | -5.658476 | -0.707501 | 2.844017 |
| H | -6.556374 | -1.231564 | 5.003518 | H | -6.621795 | -1.262548 | 4.996623 | H | -6.780283 | -1.280290 | 4.988947 |
| H | -8.884747 | -2.065719 | 5.138844 | H | -8.938312 | -2.134750 | 5.089599 | H | -9.111984 | -2.115940 | 5.001518 |
| H | -10.236581 | -2.365542 | 3.076731 | H | -10.256360 | -2.432794 | 3.005647 | H | -10.361425 | -2.394800 | 2.873141 |
| H | -7.691918 | -0.991689 | -2.557471 | H | -7.657638 | -0.949394 | -2.576526 | H | -7.543358 | -0.964559 | -2.615862 |
| H | -9.928893 | -1.706689 | -3.384727 | H | -9.872690 | -1.686025 | -3.442669 | H | -9.736815 | -1.670509 | -3.559270 |
| H | -11.673832 | -2.451645 | -1.791893 | H | -11.625880 | -2.480903 | -1.883498 | H | -11.557328 | -2.432482 | -2.062087 |
| H | -11.216263 | -2.494829 | 0.648440 | H | -11.199521 | -2.551482 | 0.561806 | H | -11.220296 | -2.501626 | 0.397208 |
| H | -6.911798 | -3.566415 | -0.166470 | H | -6.882459 | -3.565850 | -0.105850 | H | -6.874562 | -3.575665 | -0.086757 |

**Supplementary references**

[S1] K. Masui, H. Nakanotani, C. Adachi, *Org.* Electron. **2013**, *14*, 2721.
